# Views and Experiences of Persons with Chronic Diseases about Strategies that Aim to Integrate and Re-Integrate Them into Work: A Systematic Review of Qualitative Studies

**DOI:** 10.3390/ijerph15051022

**Published:** 2018-05-18

**Authors:** Eva Esteban, Michaela Coenen, Elizabeth Ito, Sonja Gruber, Chiara Scaratti, Matilde Leonardi, Olga Roka, Evdokia Vasilou, Amalia Muñoz-Murillo, Carolina C. Ávila, Dare S. Kovačič, Ivana Ivandic, Carla Sabariego

**Affiliations:** 1 Department of Medical Information Processing, Biometry and Epidemiology (IBE), Chair for Public Health and Health Services Research, Research Unit for Biopsychosocial Health, Ludwig-Maximilians-Universität (LMU), 81377 Munich, Germany; michaela.coenen@med.lmu.de (M.C.); elizabeth.ito.88@gmail.com (E.I.); ivana.ivandic@med.lmu.de (I.I.); carla.sabariego@med.lmu.de (C.S.); 2 Disability and Diversity Studies, Carinthia University of Applied Science (CUAS), 9020 Klagenfurt, Austria; s.gruber@fh-kaernten.at; 3 Neurology, Public Health and Disability Unit, Neurological Institute Carlo Besta IRCCS Foundation, 20133 Milan, Italy; chiara.scaratti@istituto-besta.it (C.Sc.); matilde.leonardi@istituto-besta.it (M.L.); 4 Department of Special Education, University of Thessaly, 38221 Volos, Greece; olgaroc1@yahoo.gr (O.R.); eudokia1@hotmail.com (E.V.); 5 Research Unit, Parc Sanitari Sant Joan de Déu, Universitat de Barcelona, Sant Boi de Llobregat, 08830 Barcelona, Spain; a.munoz@pssjd.org; 6 Department of Psychiatry, Universidad Autónoma de Madrid and Institute of Health Carlos III, CIBER of Mental Health (CIBERSAM), 28038 Madrid, Spain; carolina_avila@hotmail.com; 7 Development Center for Vocational Rehabilitation, University Rehabilitation Institute, Republic of Slovenia, 1000 Ljubljana, Slovenia; dare.kovacic@ir-rs.si

**Keywords:** employment, chronic disease, systematic review, qualitative studies

## Abstract

The effectiveness of strategies targeting professional integration and reintegration strongly depends on the experiences of participants. The aim of this systematic literature review is to synthesize European qualitative studies exploring views and experiences of persons with chronic conditions regarding strategies for integration and reintegration into work. The systematic search was conducted in Medline, PsycINFO, CDR-HTA, CDR-DARE and Cochrane Systematic Reviews. Overall, 24 studies published in English between January 2011 and April 2016 were included. Most studies were carried out in Nordic countries or in the UK, and most participants were persons with either mental or musculoskeletal disorders. Ten themes emerged: individual and holistic approach, clarity of strategy and processes, timing of rehabilitation processes, experience with professionals, at the workplace and with peer groups, changes in the understanding of health and work, active involvement in the process, competencies development and motivating aspects of work. Findings highlight, among others, the need to actively involve participants in the return to work process and to provide timely and clearly structured processes and interventions. This review provides stakeholders key information to develop, plan, implement and evaluate interventions to integrate and re-integrate persons with chronic conditions into work in Europe.

## 1. Introduction

There are plenty of strategies in European countries which aim to integrate and re-integrate persons with chronic diseases (PwCDs) in work ranging from systemic approaches to individualized and person-centered strategies [1]. As proposed by Scaratti and colleagues, these strategies can be classified at three levels, “Policies”, “Systems”, and “Services”, all with the ultimate goal to directly or indirectly contribute to the employability of PwCDs [1]. At policy level, there are national strategies such as the “Five Year Forward View for Mental Health” in the UK [2] and European strategies such as the “European Disability Strategy 2010–2020” aiming to empower PwCDs “so that they can enjoy their full rights, and benefit fully from participating in society and in the European economy (…)” ([3] p. 4) to finally implement the UN Convention on the Rights of Persons with Disabilities [4]. Examples of strategies at system level are disability benefit and pension programs implemented in several European countries and the incentive-based program Work assessment allowance (Arbeidsavklaringspenger) established in Norway [5]. At service level, there are job placement services such as the program of the Spanish Association Against Cancer targeting the specific needs of persons with cancer [5].

Strategies promoting the integration and re-integration into work for PwCDs are of utmost importance nowadays, especially in European countries with a large share of adults of working age living with chronic health conditions. European countries face the challenge of an ageing population and, more specifically, a substantially high proportion of employees aged 50 and above [6,7]. Meanwhile, the prevalence of chronic health conditions is rising [8,9,10] and a substantial proportion of persons living with chronic diseases face more than one chronic health condition at the same time [11]. There is no doubt that for persons of working age this is associated with a high burden on employers, employees and, in the long run, on the social and welfare systems of countries in which they live. At the same time, unemployment rates of PwCDs are considerably high [12,13,14,15,16,17] and, for those who do work, income is lower as compared to persons without chronic diseases [16,18,19,20]. Work, as an important life domain, has a positive impact on health and functioning in PwCDs [21,22,23] and an important share of PwCDs who are unemployed due to health-related problems wish to return to work and regain participation in this important life domain [24,25].

To overcome this contradiction, the European Union has invested considerable resources in the field. A considerable number of strategic papers and reports are stressing the need to foster employability of PwCDs [26,27,28]. The EU-funded project Participation to Healthy Workplaces and Inclusive Strategies in the Work Sector (PATHWAYS) faced the challenge to provide evidence-informed recommendations regarding strategies for the integration and re-integration of PwCDs into work [29]. Strategies to integrate and re-integrate PwCDs into work are of complex nature. At an individual level, they encompass several actors, such as the person him-/herself, his/her family, employers, co-workers, health professionals or social workers. At the system level, diverse institutions and agencies are involved, such as health and social ministries, as well as employment agencies. The effectiveness and uptake of interventions carried out in complex systems are strongly influenced by several factors, such as the quality and level of coordination and interaction of the involved actors, the social security systems of countries and the current laws. It is therefore important that stakeholders in charge of developing, planning and implementing interventions in real-life settings consider relevant factors. The views and experiences of the PwCDs themselves about strategies and programs aiming to integrate and re-integrate them into work are especially important to consider as they influence willingness and motivation of PwCDs to engage with and promote the sustainability of achieved results.

In two recently published systematic reviews, the authors found evidence supporting the effectiveness of a range of strategies for integration and re-integration into work for PwCDs in European countries [30,31]. To complement and enrich these findings, the views and experiences of PwCDs have also been taken into account by summarizing qualitative studies. The aim of this systematic literature review is therefore to assess and synthesize qualitative studies exploring the views and experiences of PwCDs regarding strategies to facilitate and manage their integration and reintegration into work life with a particular focus on aspects of the strategies that act as barriers or facilitators in the implementation and impact of these measures.

## 2. Materials and Methods

This systematic literature review was carried out within the scope of the EU-funded project PATHWAYS (Participation to Healthy Workplaces and Inclusive Strategies in the Work Sector; grant agreement n. 663474) [29]. This three-year project aimed to identify strategies supporting professional integration and reintegration for PwCDs in Europe, to evaluate their effectiveness and to assess the specific employment-related needs of these persons. The final result of PATHWAYS was to develop evidence-informed European policy recommendations to support the implementation of effective professional integration and reintegration strategies for PwCDs [32]. Partners from ten European countries, namely Austria, Belgium, Czech Republic, Germany, Greece, Italy, Norway, Poland, Slovenia and Spain, built the PATHWAYS Consortium. Within the project, a comprehensive systematic literature review was carried out to get first-hand evidence on the effectiveness of integration and reintegration strategies into work for PwCDs in European countries. With this paper, we solely focus on the studies retrieved from this systematic review using qualitative methods. For data synthesis of these qualitative studies, we used the methodology developed by the Cochrane Qualitative Research Methods Group for searching for qualitative evidence [33] presented also in the work of Ames and colleagues [34]. Results of the studies using quantitative methods are reported elsewhere [30,31]. To facilitate the comprehensiveness of the methods used in this review, we first present tasks done in the scope of the entire systematic review performed in PATHWAYS; afterwards, we indicate which additional exclusion criteria were applied to select studies for the present review.

### 2.1. Search Strategy for the Complete PATHWAYS Review

In April 2016, we ran systematic searches for the entire review in the databases MEDLINE, PsycINFO, CDR-HTA, CDR-DARE and Cochrane Systematic Reviews. For each database, search strategies were developed for studies focusing on strategies aiming at the professional integration of PwCDs. Terms to identify (quantitative and) qualitative studies were used. The search strategies are described in the Supplementary Materials. In addition, reference lists of the publications included in our review and studies included in 30 systematic reviews and meta-analyses that were retrieved from the searches were checked for papers published between 2011 and April 2016 that were not identified in the electronic searches.

### 2.2. Selection Criteria for the Complete PATHWAYS Review

Studies were included if they:

(a) had been published between January 2011 and April 2016;
(b) were published in English;
(c) were intervention studies, namely randomized trials, non-randomized controlled trials, non-controlled pre-post intervention studies; were observational studies, namely cohort studies, case-control studies, cross-sectional studies, descriptive longitudinal studies; or were qualitative studies or mixed-methods studies;
(d) had been carried out in the 28 countries of the European Union; in Norway, Lichtenstein, Iceland or Switzerland; or in non-European countries with western lifestyle: Canada, United States of America, or Australia;
(e) reported on effectiveness regarding at least one of the following work outcomes:
  - employment status (employed/unemployed);
  - return to work;
  - absenteeism (sick leave);
  - maintain a job;
  - obtain a job;
  or investigated variables potentially affecting effectiveness (e.g., views and experiences of involved persons with a given strategy);
(f) focused on the working population aged 16 to 65 years; and
(g) focused on one of the following health conditions:
  - PwCDs in general, i.e., specific conditions are not further specified in the studies or results for different conditions are reported together, and persons with disabilities in general (persons with disabilities were included because people who receive disability benefits usually have chronic diseases and experience significant levels of disability in daily life) [35];
  - mental disorders, musculoskeletal disorders, cancer, neurological, metabolic, respiratory and cardiovascular diseases; or
  - depression, back and neck pain, migraine, diabetes mellitus, chronic obstructive pulmonary disease and ischemic heart disease.

Studies were excluded if they:

(a) were published before 2011;
(b) were published in other languages than English;
(c) were case report/case series, psychometric studies, letters, comments, editorials, overviews without empirical primary or secondary data, reviews (systematic and non-systematic reviews, health technology assessments) and meta-analyses, protocols, studies reporting exclusively on design or baseline data;
(d) had been carried out in other countries than those specified above;
(e) considered neither effectiveness outcomes (for example, studies reporting only on costs resulting from the implementation of strategies) nor variables potentially affecting effectiveness;
(f) included participants mostly aged <16 or >65 years;
(g) included participants with mainly chronic diseases other than the ones defined above;
(h) did not focus on a concrete strategy or group of strategies (for example, studies focusing on general factors that facilitate return to work after sick leave); or
(i) had no abstract available.

### 2.3. Additional Selection Criteria for the Present Review

We only considered studies: (1) carried out in the 28 countries of the European Union, in Norway, Lichtenstein, Iceland or Switzerland; (2) using qualitative methods to collect and analyze data; and (3) reporting on the experience of persons with chronic conditions with a concrete professional integration strategy. Studies were excluded if it was not possible to differentiate results obtained with qualitative methods and with quantitative methods, and if we could not clearly identify the views reported by PwCDs themselves.

### 2.4. Eligibility Assessment

We retrieved all references identified in the electronic databases into one database and removed duplicates. The remaining references were distributed between five centers participating in the study. Trained reviewers screened by title and abstract to identify potentially relevant studies. At least 20% of these references were screened independently by two researchers in each center. The full-texts of the references selected in this step were assessed independently by two researchers for fulfilment of the inclusion criteria. Disagreements were solved by discussion and, if necessary, by involving a third researcher. In this step, we retrieved studies for this paper using qualitative methods. Studies using mixed methods were included if it was possible to clearly differentiate the information collected and analyzed using qualitative methods from quantitative data and results. Only those studies whose participants actively took part in or were exposed to a measure to facilitate or regulate their participation in work life were eligible for further analysis. Finally, studies were eligible if they focused on the views and experiences of PwCDs regarding concrete professional integration strategies. We excluded studies if it was not possible to assign the reported views unequivocally to the participating PwCDs.

### 2.5. Data Extraction and Methodological Assessment

The main characteristics of the included studies (reference of the publication, strategy focused, study population, context, purpose of the study, methods, main results, and limitations as stated by the authors) were compiled. One author extracted the characteristics while a second checked this data. Then, two authors re-read the Results sections of all included papers and extracted all aspects related to a professional integration measure that was associated with a positive or negative experience by the participants of the studies. This information was extracted by one author and checked by the first author. Where necessary, the extraction was discussed, completed and corrected.

We used the Consolidated Criteria for Reporting Qualitative research (COREQ [36]) and the Quality appraisal checklist for qualitative studies developed by the National Institute for Health and Care Excellence (NICE [37]) to assess the methodological quality of included studies, as well as the adaptation of the CASP checklist [38] used by Ames and colleagues [34]. COREQ consists of 32 items that covers aspects of a qualitative study that should be reported (research team, study methods, context of the study, findings, analysis and interpretations). The 12-item NICE checklist provides a more concise assessment of the study quality. To summarize the assessment, the 8 items of CASP were used. To assess the quality appraisal, we established the following procedure: First, one author applied the COREQ and NICE checklists to each study. Second, another researcher checked all checklists for discrepancies and completed the adapted CASP checklist. Disagreements were resolved through discussion or by consulting a third researcher. As recommended by Ames and colleagues [34], we did not exclude any study because of methodological limitations. Limitations of studies were considered when deciding on their relative contributions to this review (see Section 2.7).

### 2.6. Data Synthesis

For data synthesis, we performed a thematic analysis using a constant comparison strategy for data extraction and synthesis as introduced by Miles and colleagues [39]. Every aspect related to a professional integration measure that was mentioned as being positively or negatively experienced by the study participants was considered a “meaning unit”. In the data synthesis across the included studies, codes, categories and subcategories were created in an iterative process. First, two authors assigned codes to an initial set of meaning units. After discussing and agreeing on an initial set of codes, the two researchers reviewed the meaning units that were already labeled and coded further meaning units by refining codes or adding new ones. The codes and their similarities and differences were discussed on a regular basis. Categories emerged after grouping similar codes and overarching categories were identified by compiling categories in a meaningful way. Categories were considered “findings” and overarching categories were considered “themes”. Original statements of the study participants (quotations in the included papers) were selected to exemplify the content of the findings.

### 2.7. Assessment of Confidence of Findings

To assess the confidence in the review findings, we applied the GRADE-Confidence in the Evidence from Reviews of Qualitative Reviews of Qualitative research (GRADE-CERQual) [34,40]. For each finding, the following aspects were assessed:

(1) Methodological limitations of the primary studies providing data: The extent to which there are concerns about the design or conduct of these studies (for instance, concerns regarding the sampling strategy or the procedures for data collection).
(2) Coherence: Degree to which data from the primary studies support the finding (for instance, if patterns have been identified in studies with similar populations and interventions) or the finding provides a good explanation for those data.
(3) Adequacy of the data contributing to the finding in terms of richness (extent to which data provided by the studies are detailed enough to understand the phenomenon) and quantity of data (for example, if the amount of studies, settings or populations supporting the finding is considered enough).
(4) Relevance of the primary studies to the review question: The extent to which the context of the studies providing data for a given funding addresses the main components of the research question (for instance, population, setting, intervention).

Based on the assessment of these four aspects, two authors judged the overall confidence in each review funding to be [40]:

- High: It is highly likely that the review finding is a reasonable representation of the phenomenon of interest.
- Moderate: It is likely that the review finding is a reasonable representation of the phenomenon of interest.
- Low: It is possible that the review finding is a reasonable representation of the phenomenon of interest.
- Very low: It is not clear whether the review finding is a reasonable representation of the phenomenon of interest.

## 3. Results

### 3.1. Results of the Searches

**Figure 1:**
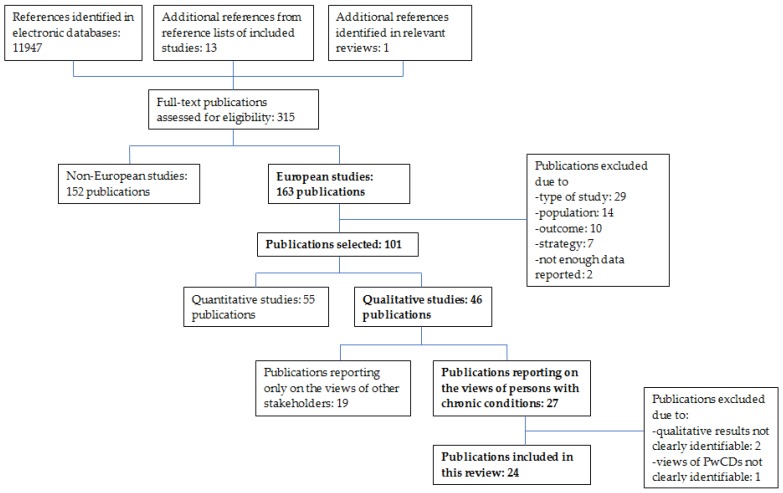
Flow chart of the entire and present systematic review according to the PRISMA flow diagram [68]; boxes in bold highlight the flow chart of the present review with its focus on qualitative studies exploring the views and experiences of PwCDs regarding strategies to facilitate and manage their integration and reintegration into work life.

The searches in the electronic databases identified 11,947 references published between January 2011 and April 2016 for the entire review (quantitative and qualitative studies). Fourteen additional documents were added; thirteen were from the reference lists of the studies included in our review and one was identified among the studies included in 30 systematic reviews and meta-analyses relevant for our research question and published in the same period. After reading the full-text of potentially relevant publications (*n* = 315) for the entire review, we identified 27 articles that met the inclusion criteria for the present review on qualitative studies. Two mixed-methods studies were excluded because it was not possible to differentiate data obtained with qualitative and quantitative methods [41,42]; a third study was excluded because we could not clearly identify the views reported by PwCDs [43]. This led to a total of 24 publications included in our review [44,45,46,47,48,49,50,51,52,53,54,55,56,57,58,59,60,61,62,63,64,65,66,67]. The flow chart of the study selection process is presented in Figure 1.

### 3.2. Main Characteristics of Included Studies

Participants in the included qualitative studies were most often persons suffering from mental disorders [44,45,46,48,50,52,56,59,60,61,62,63,67]. Three studies included persons with musculoskeletal disorders [47,51,55], two studies persons with cancer [49,66], two studies persons with mental and musculoskeletal disorders [53,64], and two studies a broad mix of persons with long-term conditions [54,65]. In the remaining two studies, participants had disabilities related to different health conditions [57,58].

In half of the studies, the professional integration strategies aimed to help unemployed persons with chronic health conditions to find a job. Studies with strategies targeting employed persons generally focused on supporting persons on sick leave to return to work. Only two studies explored a measure that tried to help workers to retain their jobs [47,48].

Thirteen studies were conducted in Nordic countries: Denmark (*n* = 4 [44,47,59,60]), Norway (*n* = 3 [53,57,61]) and Sweden (*n* = 6 [45,50,51,52,55,56]); nine studies were carried out in Belgium (*n* = 2 [66,67]), France (*n* = 1 [49]) and the UK (*n* = 6 [46,48,54,58,64,65]); and two publications reported on one study performed in different European countries, namely Bosnia, Denmark, France, Greece, Norway, Poland, Spain and the UK [62,63].

A wide range of strategies to manage professional integration were explored in the included studies, for example, collaboration strategies [44,50,59,60,61], supported employment [45,46,52,58,61], job training [57,62,63,67] and Condition Management Programs [64,65]. Three studies explored the perception of participants regarding established actors in the management of sick-leave and return to work: the general practitioner [54], the social insurance agency and health care system [55] and legislation on return to work [66]. The main characteristics of the studies included in the review are shown in Appendix A.

The quality assessment of the included studies showed, in general, restricted reporting of methodological details, probably due to word limits of the journals. All studies contained some information about setting, participants, data collection and analyses but often missed information about sampling and recruitment strategies and the context of data collection. The reporting of results varied broadly in structure and detail (e.g., depth of findings), but in most of the studies it was coherent and supported by quotations. In many studies, reflexivity of the research process was poorly discussed or not addressed.

### 3.3. Themes and Findings Identified in the Included Studies

As the result of the synthesis of the thematic analysis, 31 findings emerged from the 24 studies, which were allotted to the following 10 themes:

(1) Individual and holistic approach (3 findings)
(2) Clarity of the integration strategy and process (4 findings)
(3) Timing of the rehabilitation process (2 findings)
(4) Experience of persons with chronic diseases with the professionals (5 findings)
(5) Changes in the understanding of health and work situation (3 findings)
(6) Active involvement of the persons with chronic health conditions in process of professional integration (1 finding)
(7) Competencies developed by the participant (2 findings)
(8) Experience of participating in a group with other persons with chronic health conditions (3 findings)
(9) Experience at the workplace (3 findings)
(10) Motivating aspects of work (5 findings)

In the sections below, we briefly describe the findings for each of the ten themes. A summary of our judgement of confidence for each finding is shown in Table 1. The CERQual evidence profile supporting the assessment of confidence in each finding can be found in the Supplementary Materials.

#### 3.3.1. Individual and Holistic Approach

Finding 1: PwCDs Wanted Professionals to Show a Genuine Interest to Understand and Accept Them as Individuals (Moderate Confidence).

In some studies, participants expressed their wish to be perceived and accepted as persons with unique circumstances, experiences and needs [44,45,51,53,55,60,61,64]. Participants appreciated personal meetings where they could describe their situation and be heard and believed (“…she [the return to work coordinator] saw me as a human being and not only as a statistic. She saw me, not as a case, but as an individual who needed some kind of help.” [44]). Despite the wish to be heard, some PwCDs have difficulties explaining symptoms that could not be clearly seen and objectively measured, for example, decreased concentration and energy in persons suffering from depression or musculoskeletal pain; other subjects were afraid of intensification of symptoms such as anxiety if they had to focus on them again [44]. Some persons participating in a multidisciplinary intervention expressed the confidence that different professionals involved could inform each other and achieve a better understanding of their situation [61].

Finding 2: PwCDs Found It Helpful to Be Seen from Different Perspectives (e.g., Medical, Psychological) and in More Areas than the Ones Directly Related to Work (Moderate Confidence).

Some participants appreciated that different professionals tried to understand them from different perspectives (e.g., physician, physiotherapist and psychologist) and that other areas of life apart from work were also the object of analyses and intervention [44,51,58,59,60,61,65,67] (“And getting that support purely privately, too. How could I manage my day at all, perhaps not only at work; but there were lots of other things involved that they (the coached work-training team) had to help me with. When all that worked, my job went okay” [51]).

Finding 3: Flexibility in the Implementation of the Strategy and the Contact with the Professionals Was Highly Appreciated by the Participants (Moderate Confidence).

In many studies [44,45,48,50,51,57,58,60,61,64,65,67], participants reported that they appreciated receiving tailored support regarding:

- place, time and number of meetings with the professionals;
- rhythm of progress; and
- the kind of activities and how they were implemented.

This flexibility confirmed to participants, that their needs and preferences had been understood and taken seriously, and they found the intervention more comfortable and feasible for them (“I myself have decided when I wanted to take a step forward.” [61]). Lack of information and collaboration between involved actors were important obstacles for the adaptation of the integration measure to the individual circumstances. This was the case when the unemployment office assigned participants to jobs requiring training they lacked [51] or when the insurance office supported vocational rehabilitation for a fixed period, setting some PwCDs under time pressure [50].

#### 3.3.2. Clarity of the Integration Strategy and Process

Finding 4: Providing a Definition and Clarification of the Problem and Setting a Clear Course of Action Was Found Helpful (Low Confidence).

Two studies reported that participants appreciated having a clear and concrete plan for the steps given to improve their work situation [44,65]. Subjects had the experience of moving from a more or less chaotic situation without effective handling into purposeful, meaningful and hopeful actions (“We have got goals, not just ploughing through life aimlessly, that’s what I feel”, female, mental disorder [65]).

Finding 5: The Role of Activities and Professionals Was Sometimes Difficult to Understand, Leading to Confusion and Demotivation (Moderate Confidence).

Participants in four studies felt uncertain about the aim of some activities or the role played by some of the professionals or staff they met [44,50,60,61]. All four studies analyzed the experiences of persons with mental disorders with an intervention involving different professionals and agencies. Cognitive limitations associated with their health condition, poor provision of information to participants and organizational deficits in some institutions were identified by the authors as potential variables leading to poor understanding and demotivation.

Finding 6: Persons Found It Difficult to Move Forward Because of Receiving Wrong or Insufficient Information about Requisites, Procedures and Decisions Affecting Their Professional Integration (Moderate Confidence).

In three studies, participants stated that being insufficiently informed about their possibilities to improve their work lives hindered their initiative, sometimes because of fear of doing something wrong and losing social benefits, and made them feel frustrated and powerless to influence their lives [55,61,66].

Finding 7: PwCDs Appreciated that Professionals and Institutions Shared a Common Understanding of Their Situation and the Intervention Plan (Low Confidence).

Subjects participating in interventions that support their professional integration are usually in contact with different professionals and institutions that may influence their work integration process and financial situation (e.g., health care professionals, social insurance agencies, and employers). In three studies, participants found it important that these actors had a similar understanding of the situation and that they agreed with the plan of action and collaborated to implement it [45,50,61].

#### 3.3.3. Timing of the Rehabilitation Process

Finding 8: People Would Have Liked to Start Working for Their Professional Integration at an Earlier Stage (Moderate Confidence).

In five studies, participants expressed their wish to have started to work actively for their return to work life earlier [55,56,59,60,66]. After getting a sick certificate, some subjects experienced the period on sick leave and being treated with medicines as too long and not helpful [55,56,66]. In other cases, subjects had to delay or cancel their participation due to obstacles such as a traveling distance to the intervention activities that was too long or waiting lists [59,60] (“These were months where I really needed help…then I could have recovered much quicker” [60]).

Finding 9: A Continuous Rehabilitation Process is Positively Experienced by Subjects (Low Confidence).

Participants in three studies appreciated being involved in a continuous process without long waiting times to contact professionals, to get information and to get services [50,55,61]. Some of the reported obstacles to an ongoing and continuous process were: organizational aspects of institutions that led to frequent changes in the assigned contact person, poor collaboration between agencies (e.g., health care system and social insurance), and bureaucratic structures that make establishing and maintaining contact difficult. (“…there has been so much muddle because they [the employment office] were of the opinion that I was enlisted in a rehabilitation program and they did not want to do anything with me” [50]). A process with frequent interruptions or an arduous progression led to feelings of resignation, passivity, powerlessness and stress, worsening the health status and decreasing the engagement of the individuals.

#### 3.3.4. Experience of PwCDs with Professionals

Finding 10: Perceiving Professionals as Experts Increased the Engagement of the Participants with the Intervention (Moderate Confidence).

Individuals participating in interventions often had intensive contact with professionals to whom they had to open up and describe their experiences in detail (e.g., psychologist, case coordinator, and employment specialist). When subjects perceived these professionals as experts in their field, they developed trust and felt safe and well guided through their process [44,45,48,58,59,66]. Professionals had to convince participants about their expertise in the interaction and handling the situation; owning a title was not enough [44,58]. Individuals valued knowledge and technical skills, such as knowledge about the disease and knowing how to write a bio sketch, and pointed out how this expertise led to an interaction that made them feel comfortable (“The ES [employment specialist] realizes how I have been coping all these years, and has knowledge about my difficulties. I do not have to worry about a lot of tricky questions…I don’t need to be afraid or show my claws as a way of defending myself.” [45]).

Finding 11: The Interaction Style of The Professional Was Perceived as a Key Element of the Intervention (Moderate Confidence).

In many studies, participants pointed out the importance of the quality of the personal meeting with the specialists involved in their professional integration [44,48,51,55,60,61,64,65,66]. The ability of the professional to listen, ask helpful questions, provide feedback, show empathy, and be respectful and positive communicated genuine interest, acknowledgement and esteem. The participant developed trust and was more open to share information about experiences, needs and difficulties (“a very good listener” (…) “very, very helpful” (…) “a very calming and soothing influence”; “I feel really at ease with [Retain-project worker]. (She)’s the only person I’ve told everything to…I trust her” [48]).

Finding 12: Participants Appreciated Receiving Practical Support (Moderate Confidence).

Participants generally appreciated having contact with supportive professionals who were not just administrative intermediaries but provided practical support that was related to work issues, general management of disease and private aspects of life [45,46,48,51,54,57,58,59,61,65,66]. Receiving support in certain areas was particularly important, i.e., where disequilibrium in hierarchy and power or the existence of bureaucracy made access or collaboration difficult for the subject: for example, in meetings with authorities and with the employer and in helping to get funding and sick certificates.

Finding 13: Stability and Availability of the Support Person Was Associated with a Sense of Security and Relief (Moderate Confidence).

Some participants mentioned the positive effect of feeling that there was someone ready to hear and help them whenever necessary, while frequent changes in the reference or contact person or difficulties to be in contact were experienced as stressful [58,61,65].

Finding 14: Professionals Were a Source of Emotional Support for Participants (Moderate Confidence).

Some subjects felt that the professional with whom they had the most contact motivated them and built their self-confidence through a positive attitude, expressions of confidence and interest in other areas in addition to working life [45,46,48,57].

#### 3.3.5. Changes in the Understanding of Health and Work Situation

Finding 15: Participants Liked Increasing Their Understanding of Symptoms and Mechanisms of the Disease, and Their Health Status (High Confidence).

An increased knowledge about the disease and its interaction with different variables helped participants to feel less upset about the health situation. Many subjects changed from being overwhelmed by their reactions and feeling like passive victims to understanding and feeling the potential to control the disease. They could provide professionals with more relevant information (e.g., about risky situations), their motivation to collaborate and follow the recommendations of the experts increased, and they identified new potential ways to help themselves [44,45,47,49,56,64,65].

Finding 16: Professionals’ Focus on Workplace Conditions Helped Participants to Broaden Their Understanding of Work Dynamics and Showed Them Possible Ways to Be a Resource for the Company (Moderate Confidence).

Some studies reported how counselors and other professionals focused on the role played by workplace aspects, analyzed the PwCD’s concrete situation together with the participant and identified work conditions that could facilitate return to work and job maintenance. Subjects understood the importance of discussing possible accommodations with the employer and learned to see themselves as a resource at work, given supportive conditions [47,48,57]. “I can you know remind me ‘….you are an asset to your company if it’s done in the right way and you’re not under too much pressure’. She [retain project worker] really made me see that and it’s amazing because it turned around my view of the situation” [48].

Finding 17: Participants Appreciated Increasing Their Own Understanding (Moderate Confidence).

For some participants, it was important to reflect on their preferences and values and learn about their strengths and weaknesses [48,53,57,62]. Reviewing values helped them to clarify the meaning of work in their lives and set their own relevant goals. Participants felt more motivated to invest energy in a course of action that was meaningful for them. Identifying their positive achievements and skills increased their self-esteem and allowed them to focus on their own resources and possibilities.

#### 3.3.6. Active Involvement of the Persons with Chronic Health Conditions in Process of Professional Integration

Finding 18: Participants Appreciated Working Actively and Being Involved in the Development and Implementation of The Activities to Improve Their Professional Integration (Moderate Confidence).

Some studies reported that participants liked to play an active role in their process of work integration [45,47,51,56,61,65]. Participants described how professionals recognized them as reliable informants to describe difficulties and legitimate partners to set goals and plan activities as well as how they were helped to manage themselves. For example, instead of advocating for a silent employee in meetings with the employer, professionals helped participants to clarify what they wanted to say and to communicate it effectively [48]. This focus on personal initiative and efforts helped many PwCDs to move from a more passive sick-role to a status of “actor”.

#### 3.3.7. Competencies Developed by the Participant

Finding 19: Participants Welcomed Being Taught Skills to Manage Problematic Situations in Different Areas of Life (Moderate Confidence).

Learning strategies to effectively manage the concrete situations faced by the participants was a component of almost all interventions and was appreciated by the participants [46,48,51,56,59,60,61,63,64,65]. In some cases, the focus was on handling the challenges of the workplace [48,51,56,57,60,62] or the management of the disease [59,64]. Participants were also taught strategies for managing life in a more general sense, for example, developing communication skills, applying problem solving and learning to focus and set attainable goals [46,51,61,63,65]. After a long period of sick-leave, some people learned to introduce routine and regularity into daily life [46,49]. An important aspect of skill development was “learning by doing” and practicing and learning the skills in the real situation whenever possible.

Finding 20: Participants Felt that Being Immersed in a Social Atmosphere Offered Them the Opportunity to Develop or Practice Social Skills (Low Confidence).

Being in a social context (training courses, practical placement or work) allowed for getting used to social interaction after a long sick leave or unemployment phase, practicing or developing social skills [46,53,57].

#### 3.3.8. Experience of Participating in a Group with Other Persons with Chronic Health Conditions

Finding 21: Several Subjects Attending an Educational or Support Group Found That Meeting Other Persons Who Faced Similar Problems Made Them Feel “Normal” and Less Isolated (Moderate Confidence).

Meeting persons facing a similar situation and talking with them about their experiences showed them that they were not the only person living with their symptoms and work life problems. Participants felt “normal” and less bothered, developed a sense of companionship and security and increased their self-confidence [44,48,49,51,53,56,65]. “I could see how other people coped with the disease”, “I felt normal” [49].

Finding 22: Meeting Persons with Similar Concerns Helped Participants to Learn More about Their Situation and Motivated Them to Try a New Approach (Moderate Confidence).

Participants realized there were common aspects between their experience and that of others; by hearing them, they increased their understanding of their own feelings and reactions, as well as those of others, and the variables involved in their situation. They exchanged and developed strategies to manage their life and reinforced each other by acknowledging their steps forward [49,53,56,65].

Finding 23: Some Persons Experienced Difficulties Sharing Personal Experiences in a Group (Low Confidence).

Participating in a group was not a positive experience for all subjects. Some people had some inhibitions, particularly at the beginning, regarding talking about sensitive things and pointed out the importance of assuring confidentiality [56,64]. Differences in motivation and positive attitudes among participants caused problems for some subjects who had less energy and hope and felt like “losers” [53].

#### 3.3.9. Experience at the Workplace

Finding 24: Support from Employers and Managers Is Perceived by Employees as a Key Element to Succeed in Their Professional Integration (Moderate Confidence).

Support from authority persons at work (i.e., employer and prime line manager) was considered a determinant of successful work resumption and maintenance, even more important than support from colleagues [48,51,53,66]. Making possible accommodations was an important way to provide support. Adaptation of the job conditions included schedule flexibility, reducing working days, changes in tasks, changes in workload and physical adaptations [48,56,61]. Persons with chronic conditions also emphasized the importance of perceiving good will, genuine interest, and understanding and respectful treatment by the employer or manager [45,48,51,56,58] (“I had a fantastic employer who paved the way for my return. He asked me if I could do this or that. They were fantastic at the workplace. They put so much effort in keeping me so I got a lot of confidence—when they take the chance I have to do my bit.” [53]).

Finding 25: The Amount of Support Provided by Colleagues Plays an Important Role in Work Integration (Moderate Confidence).

In three studies, participants pointed out that the existence or absence of support from colleagues affected work resumption and maintenance [48,51,58].

Finding 26: Some Organizations Have a Working Culture and Physical Environment that Makes the Provision of Support Easier (Moderate Confidence).

Organizations that had a flexible and accommodating physical environment (e.g., adjustable tables) and working philosophy (e.g., flexible schedules and working from home), respectful and collaborative staff interaction and skilled managers could easily provide support to persons with chronic health conditions [56,57,62]. Some employees and trainees who did not feel treated as equals and felt stigmatized left the company or were on sick-leave longer [48,58,67]. “I was off work for nearly four months through bullying and intimidation…so I went through like hell in that four months; very stressed and nearly I ended up going down on the depression route but luckily I managed to pick meself up. [Support worker] supported me with every single meeting that I had with the council, with HR and me overall boss and me union to try and get it all sorted so I could go back to work.” [58].

#### 3.3.10. Motivating Aspects of Work

Finding 27: The Expectation to Improve Their Financial Situation Motivated People with Chronic Health Conditions to Find a Job (Moderate Confidence).

In four studies, the prospect of improving their financial situation was one of the main aspects that motivated unemployed persons to find a job [52,58,61,67]. Very low wages and the risk of worsening their financial situation by losing benefits that were not compatible with work were associated with a decrease of interest in the work integration strategy [52,67].

Finding 28: Subjects Found that Having a Job or Doing an Internship Facilitated Social Inclusion and Interaction (Moderate Confidence).

Some participants reported that being active after a period of unemployment increased their social interaction through contact with colleagues, customers and other groups, and offered them the chance to integrate in a social context (“You are part of something, you have a job to talk about” [57]).

Finding 29: Having an Occupation Allowed Persons with Chronic Diseases to Focus on Something Other than the Disease (Low Confidence).

In a few studies, unemployed persons suffering from a mental disorder reported that work could help them think about something other than the disease by giving more structure in everyday life and keeping them busy [45,61,63].

Finding 30: Participants Found It Very Important to Do Something that They Could Enjoy (Very Low Confidence).

In two studies, participants stressed the relevance of having an occupation that they enjoyed as well as learning and training in something they liked [46,63].

Finding 31: Having a Job Was Associated with an Increase in Self-Esteem and Self-Confidence (Low Confidence).

The positive impact of work on affect was reported in three studies [58,61,62]. Participants who were active after a period of unemployment indicated how work improved their self-esteem and self-confidence (“I feel like a completely different person since I came to work here, I’m much happier and more confident” [58]).

## 4. Discussion

This systematic literature review assessed and synthesized qualitative studies exploring the views and experiences of PwCDs in European countries regarding strategies to facilitate and manage their integration and reintegration into work. We analyzed and synthesized data retrieved from 24 studies published in English between January 2011 and April 2016. The major share of these studies was carried out in Nordic countries. The participants of the included studies were mainly persons with mental disorders, followed by persons with chronic musculoskeletal disorders. Ten themes emerged from the included studies: “Individual and holistic approach”, “Clarity of the integration strategy and process”, “Timing of the rehabilitation process”, “Experience of persons with chronic diseases with the professionals”, “Changes in the understanding of health and work situation”, “Active involvement of the persons with chronic health conditions in process of professional integration”, “Competencies developed by the participant”, “Experience of participating in a group with other persons with chronic health conditions”, “Experience at the workplace”, and “Motivating aspects of work”.

Our review synthesizes, from the point of view of the users, aspects of strategies to integrate or reintegrate PwCDs that might be associated to their positive or negative effects. Data synthesis of the studies revealed that experiences such as feeling perceived and accepted as a person with individual needs [44,45,51,53,55,60,61,64], being treated with respect and being taken seriously are of utmost importance for PwCDs [44,45,51,53,55,60,61,64] during the process of integration and reintegration into work. In addition, PwCDs frequently report that having a voice with regard to formal arrangements such as deciding on time and frequency of appointments or planning of activities is important [44,45,48,50,51,57,58,60,61,64,65,67]. They also appreciate being actively involved in the process instead of being “treated or helped” with passive measures [55,56]. A timely start and continuous overall process of integration and of individual integration interventions are also perceived as supportive [55,59,60,61]. A delayed start of interventions and repeated waiting times in the process are negatively experienced by PwCDs [55] and evoke feelings of helplessness and passivity [61]. The presence or lack of coordinated strategies on the part of involved agencies is also perceived as a positive and negative experience, respectively [50,51]. Thus, it becomes evident in some of the studies that one agency involved in the process should know what another is going to do and should take into account formal regulations; time frames must be known and agreed upon by all agencies and professionals involved in the process and decisions have to be communicated to PwCDs in a timely manner and agreed upon with the PwCD [45,51]. In addition, positive attitudes of employers and their willingness to indeed implement accommodations contribute to the effectiveness of strategies from the perspective of PwCDs [45,48,51,53,56,58,61,66]. These and other experiences and how they are perceived by PwCDs will determine whether a strategy or program will be accepted. Therefore, studies about the effectiveness of strategies and programs for integration and reintegration into work should ideally be complemented by qualitative studies focusing on views and experiences of PwCDs. With the conceptualization and work done in the PATHWAYS project, we follow this approach, combining systematic literature reviews [30,31] with qualitative studies [69] to gain evidence and first-hand data from the field.

The context in which strategies aiming to integrate and reintegrate PwCDs into work are implemented, especially the welfare model of the country or region, is of outmost importance for the appraisal of the results of this study. The major share of studies included in this review was conducted in Nordic countries (Denmark, Sweden, and Norway). Nordic countries are characterized by welfare models that share an emphasis on egalitarianism and universal welfare provision [70], the provision of universal and generous benefits and a strong redistributive social security system [71,72], as well as active labor market policies and strong employment orientation [73]. In recent years, several policy and system-level changes have been introduced in these countries, such as partial sick leave and flexible jobs (flex-jobs) [74,75], to overcome limited financial resources. Most of the remaining studies were performed in the UK. The UK is characterized by a welfare model that emphasizes activation measures [73], a low level of government spending on social protection and modest benefits [72,76], as well as little redistribution of incomes [72]. During the last decade, several innovative changes have been implemented in the UK such as the “Statement of Fitness to Work” (“Fit Note”) for persons requiring time off of work or adaptations to their work due to illness [77] and “Individual Placement and Support”, an evidence-based approach to employment support for people with severe mental disorders [78]. What Nordic countries and the UK have in common is the acknowledgment of the need to bring PwCDs back to work, considering the importance of work for persons’ physical and mental health as well as (full) participation in society [79]. The experiences of these countries are a valuable resource for other European countries. However, it remains unclear whether the programs and strategies implemented in Nordic countries and the UK are transferable to other countries and, more specifically, other European welfare models (e.g., Mediterranean or Continental model) in times where financial resources are limited and must be invested in cost-effective interventions.

Almost all studies identified and synthesized in this review focus on strategies tailored to people with mental or musculoskeletal disorders, which are the two health condition groups that contribute the most to sick-absences and disability pensions in European countries [80,81,82,83,84,85]. Both health condition groups are characterized by non-visible and “subjective” symptoms such as pain and difficulties in emotional and cognitive functions. It is particularly important for persons with these health conditions to be believed and taken seriously when describing and reporting their symptoms and complaints, which was one of the key experiences that participants in the studies included in our review reported. We cannot conclude from our results that these experiences are also of relevance for persons with other health conditions such as metabolic or neurological diseases. However, we know from the study performed by Foitzek and colleagues that explored the needs of persons affected by a wide range of chronic diseases that being believed and recognized as an individual with specific impairments and limitations is also important for persons with other health conditions [69]. We also have to take into account when interpreting our results that there are health conditions such as cancer for which far-reaching medical innovations have been developed and established in the last decade(s). For a major share of working-aged persons diagnosed with cancer, it is possible nowadays to return to work. However, we found only limited evidence on the experiences and views of these persons.

The study populations of the studies included in this review are made up of persons who participated on a voluntary basis in these studies and were “objectively” identified as having a chronic health condition, for example, through sick leave certificates. In addition, many studies did not describe the sample selection procedure or the characteristics of the participants well enough, thus hindering conclusions about the participants in the qualitative studies. It remains unclear whether persons not involved in these studies share the same experiences. There are also persons with health conditions that are characterized by changeable symptoms with regard to time and severity, such as migraine or multiple sclerosis, that were not considered in the studies included in this review. It is an open question whether persons affected by these health conditions would also benefit from work integration strategies in a comparable way and how they would experience these strategies and interventions. Here, we assume that targeting employers and providing knowledge about the disease, the possibility and necessity to accommodate, having days off [69] and providing information about best-practice examples implemented in companies might be helpful to reduce stigma and increase openness in society, particularly in workplaces. Consequently, this would also promote persons’ self-disclosure of the disease at the work place [86,87], which is known to be associated with improving self-efficacy and disease-related self-management activities [88].

European stakeholders are currently looking for innovative strategies to improve the integration and reintegration of PwCDs in work. We know from several studies that PwCDs want to work or want to return to work [45,57,58,61,63]. Gaining work or being involved in work are important aspects of people’s lives and contribute to the financial security of persons and their relatives as well as social participation. Having a job is usually related to a clearly structured daily routine and can give a person something to talk about and share with others at the workplace and beyond. This review informs stakeholders about key elements of experiences and views of PwCDs which must be taken into account when implementing strategies and programs supporting professional integration and reintegration of PwCDs with the final goal of being effective and well-accepted. With its focus on qualitative studies this review complements existing reviews [1,30,31] and policy recommendations [32].

Many of the studies included in our review did not report much information on the background of the persons recruited for the study and collecting and analyzing data, and most studies did not discuss the potential influence of the researchers’ positions. The views and values of the person selecting and contacting potential participants may influence who is considered an “adequate” subject for the study, as well as the decision of the person to participate or not. For example, it can make a difference if the recruiter is a social insurance officer or the support person with whom the subject has a more personal and intensive contact.

Regarding the research team completing the present review, all authors involved in the assessment and analysis of qualitative data have experience in the field of disability and chronic health conditions. E.E., M.C., C.S. and I.I. are psychologists; E.I. is an occupational therapist; and S.G. is a sociologist. We share a public health perspective but believe that the effective design and implementation of strategies at the population level require an understanding of the real views and experiences of all stakeholders involved (e.g., participants, employers, and policy makers). We were aware of our backgrounds when extracting and analyzing data; we discussed our work regularly and reviewed our analyses after receiving complementary or alternative information.

There are some limitations of the present work that should be mentioned. First, we included scientific publications published between 2011 and 2016. This short five-year period was selected because of the conceptualization of the PATHWAYS project, which aims to provide an overview of the research in various disease groups, countries and different kinds of strategies. Due to feasibility reasons we had to limit the period of our searches for this review. On the same token, in the PATHWAYS project, we searched for specific health conditions, selected because of their relevant impact on labor market participation in the European context. Including more disease groups and specific conditions would have been desirable but was not feasible in the scope of the project. Second, we limited our searches to studies published in the English language and performed in European countries. We are aware that there might be studies of interest that have been published in the languages of the countries implementing them. Third, extracting information from the included qualitative studies proved to be difficult because studies and their reporting differed substantially in the level of detail of the methods used and the granularity of results reported. We used different strategies to overcome these differences in the reporting of the studies and to assure standardization of data extraction and building of categories and themes. In the future, we highly recommend implementing reporting guidelines such as COREQ when drafting qualitative papers. We know that word limits of scientific journals often contradict the application of reporting guidelines in qualitative studies. However, editors and researchers should be encouraged to go beyond established scientific conventions to facilitate the publication of qualitative studies and ensure their trustworthiness. Fourth, the development of categories and themes were conducted by four researchers and discussed with a fifth researcher. Researchers involved in this task had different professional backgrounds which enrich our data synthesis. Other researchers might have coded and extracted information in another way. To minimize the bias during the data extraction and synthesis, we established various strategies, such as the iterative process of coding, discussions between the researchers involved in the synthesis and structured discussions in case of disagreements.

## 5. Conclusions

This review synthesized 24 qualitative studies exploring the views and experiences of PwCDs in European countries regarding strategies to facilitate and manage their integration and reintegration in work life. The major share of these studies was carried out in Nordic countries and the UK and focused mainly on persons with mental and musculoskeletal disorders. We synthesized several themes highlighting the need to actively involve PwCDs in the return to work process and to provide timely and clearly structured processes and interventions. Results of this review will provide stakeholders with evidence regarding which factors must be considered when developing, planning, implementing and evaluating interventions to integrate and re-integrate persons with chronic conditions at work in Europe.

## Figures and Tables

**Table 1:** Summary of qualitative findings.

Findings		Confidence of Findings *	Explanation for Level Confidence	Contributing Studies
**Use of an individual and holistic approach**				
1.	Persons with chronic health conditions wanted professionals to show a genuine interest to understand and accept them as individuals	Moderate	Moderate concerns about methodological limitations; moderate concerns about relevance	Andersen 2014 [44], Areberg 2013 [45], Glavare 2012 [51], Haugli 2011 [53], Hubertsson 2011 [55], Martin 2012 [60], Mikkelsgard 2014 [61], Reagon 2011 [64]
2.	Persons with chronic conditions found it helpful to be seen from different perspectives (e.g., medical, psychological) and in more areas than the one directly related to work	Moderate	Moderate concerns about methodological limitations; moderate concerns about relevance	Andersen 2014 [44], Glavare 2012 [51], Lewis 2013 [58], Martin 2012 [60], Martin 2015 [59], Mikkelsgard 2014 [61], Secker 2012 [65], Vandekinderen 2012 [67]
3.	Flexibility in the implementation of the strategy and the contact with the professionals was highly appreciated by the participants	Moderate	Moderate concerns about methodological limitations; moderate concerns about relevance	Andersen 2014 [44], Areberg 2013 [45], Cameron 2012 [48], Germundsson 2011 [50], Glavare 2012 [51], Kalef 2014 [57], Lewis 2013 [58], Martin 2012 [60], Mikkelsgard 2014 [61], Reagon 2011 [64], Secker 2012 [65], Vandekinderen 2012 [67]
**Clarity of the integration strategy and process**				
4.	Providing a definition and clarification of the problem and setting a clear course of action was found helpful	Low	Minor concerns about methodological limitations; moderate concerns about relevance and adequacy	Andersen 2014 [44], Secker 2012 [65]
5.	The role of activities and professionals is sometimes difficult to understand, leading to confusion and demotivation	Moderate	Moderate concerns about methodological limitations and relevance	Andersen, 2014 [44], Germundsson, 2011 [50], Martin, 2012 [60], Mikkelsgard, 2014 [61]
6.	Some persons found it difficult to move forward because of receiving wrong or insufficient information about requisites, procedures and decisions affecting their professional integration	Moderate	Moderate concerns about methodological limitations, relevance and adequacy	Hubertsson 2011 [55], Mikkelsgard 2014 [61], Tiedtke 2012 [66]
7.	Persons with chronic diseases appreciated that professionals and institutions shared a common understanding of their situation and the intervention plan	Low	Moderate concerns on methodological limitations and adequacy; substantial concerns on relevance	Areberg 2013 [45], Germundsson 2011 [50], Mikkelsgard 2014 [61]
**Timing of the rehabilitation process**				
8.	Some people would have liked to start working for their professional integration at an earlier stage	Moderate	Moderate concerns about methodological limitations; minor concerns about relevance	Hubertsson 2011 [55], Jansson 2014 [56], Martin 2012 [60], Martin 2015 [59], Tiedtke 2012 [66]
9.	A continuous rehabilitation process is positively experienced by subjects	Low	Moderate concerns about methodological limitations, relevance and adequacy	Germundsson, 2011 [50], Hubertsson 2011 [55], Mikkelsgard 2014 [61]
**Experience of persons with chronic diseases with professionals**				
10.	Perceiving professionals as experts increased the engagement of the participants with the intervention	Moderate	Moderate methodological limitations; minor concerns about relevance	Andersen 2014 [44], Areberg 2013 [45], Cameron 2012 [48], Lewis 2013 [58], Martin 2015 [59], Tiedtke 2012 [66]
11.	The interaction style of the professional was perceived as a key element of the intervention	Moderate	Moderate concerns about methodological limitations; minor concerns about relevance	Andersen 2014 [44], Cameron 2012 [48], Glavare 2012 [51], Hubertsson 2011 [55], Martin 2012 [60], Mikkelsgard 2014 [61], Reagon 2011 [64], Secker 2012 [65], Tiedtke 2012 [66]
12.	Participants appreciated receiving practical support	Moderate	Moderate concerns about methodological limitations; minor concerns about relevance	Areberg 2013 [45], Boycott 2015 [46], Cameron 2012 [48], Glavare 2012 [51], Higgins 2014 [54], Kalef 2014 [57], Lewis 2013 [58], Martin 2015 [59], Mikkelsgard 2014 [61], Secker 2012 [65], Tiedtke 2012 [66]
13.	Stability and availability of the support workers was associated with a sense of security and relief	Moderate	Moderate concerns about methodological limitations and moderate concerns about relevance	Lewis 2013 [58], Mikkelsgard 2014 [61], Secker 2012 [65]
14.	Professionals were a source of emotional support for participants	Moderate	Minor concerns about methodological limitations and moderate concerns about relevance	Areberg 2013 [45], Boycott 2015 [46], Cameron, 2012 [48], Kalef 2014 [57]
**Changes in the understanding of the health and work situation**				
15.	Participants liked to increase their understanding of symptoms and mechanisms of the disease, and their health status	High	Minor concerns about methodological limitations, coherence and adequacy	Andersen 2014 [44], Areberg 2013 [45], Buus 2015 [47], De Blasi 2014 [49], Jansson 2014 [56], Reagon 2011 [64], Secker 2012 [65]
16.	Professionals’ focus on workplace conditions helped participants to broaden their understanding of work dynamics and showed them possible ways to be a resource for the company	Moderate	Moderate concerns about methodological limitations and relevance; minor concerns regarding adequacy	Buus 2015 [47], Cameron 2012 [48], Kalef 2014 [57]
17.	Participants appreciated increasing their own understanding	Moderate	Moderate concerns about methodological limitations and relevance	Cameron 2012 [48], Haugli 2011 [53], Kalef 2014 [57], Nieminen 2012 [62]
**Active involvement of the persons with chronic health conditions in process of professional integration**				
18.	Participants appreciated working actively and being involved in the development and implementation of the activities to improve their professional integration	Moderate	Minor concerns about methodological limitations; moderate concerns about relevance	Areberg 2013 [45], Buus 2015 [47], Glavare 2012 [51], Jansson 2014 [56], Mikkelsgard 2014 [61], Secker 2012 [65]
**Competencies developed by the participant**				
19.	Participants welcomed being taught skills to manage problematic situations in different areas of life	Moderate	Moderate concerns about methodological limitations and relevance	Boycott 2015 [46], Cameron 2012 [48], Glavare 2012 [51], Jansson 2014 [56], Martin 2012 [60], Martin 2015 [59], Mikkelsgard 2014 [61], Ramon 2011 [63], Reagon 2011 [64], Secker 2012 [65]
20.	Participants felt that being immersed in a social atmosphere offered them the opportunity to develop and practice social skills	Low	Moderate concerns about methodological limitations; substantial concerns about relevance	Boycott 2015 [46], Haugli 2011 [53], Kalef 2014 [57]
**Experience of participating in a group with other persons with chronic health conditions**				
21.	Several subjects attending an educational or support group found that meeting other persons who face similar problems made them normalize their situation	Moderate	Moderate concerns about methodological limitations; minor concerns about relevance	Andersen 2014 [44], Cameron 2012 [48], De Blassi 2014 [49], Glavare 2012 [51], Haugli 2011 [53], Jansson 2014 [56], Secker 2012 [65]
22.	Meeting persons with similar concerns helped participants to learn more about their situation and motivated them to try a new approach	Moderate	Moderate concerns about methodological limitations and relevance; minor concerns regarding adequacy	De Blassi 2014 [49], Haugli 2011 [53], Jansson 2014 [56], Secker 2012 [65]
23.	Some persons experienced difficulties sharing personal experiences in a group	Low	Serious concerns about methodological limitations; moderate concerns about relevance; minor concerns about adequacy	Haugli 2011 [53], Jansson 2014 [56], Reagon 2011 [64]
**Experience at the workplace**				
24.	Support from employers and managers is perceived by employees as a key element to succeed in their professional integration	Moderate	Moderate concerns about methodological limitations and relevance	Areberg 2013 [45], Cameron 2012 [48], Glavare 2012 [51], Haugli 2011 [53], Jansson 2014 [56], Mikkelsgard 2014 [61], Lewis 2013 [58], Tiedtke 2012 [66]
25.	The amount of support provided by colleagues plays an important role in work integration	Moderate	Moderate concerns about methodological limitations and relevance	Cameron 2012 [48], Glavare 2012 [51], Lewis 2013 [58]
26.	Some organizations have a working culture and physical environment that makes the provision of support easier	Moderate	Moderate concerns about methodological limitations and relevance	Cameron 2012 [48], Kalef 2014 [57], Jansson, 2014 [56], Lewis 2013 [58], Nieminen 2012 [62], Vandekinderen 2012 [67]
**Motivating aspects of work**				
27.	The expectation to improve their financial situation motivated people with chronic health conditions to find a job	Moderate	Moderate concerns about methodological limitations and relevance	Hasson 2011 [52], Lewis 2013 [58], Mikkelsgard 2014 [61], Vandekinderen 2012 [67]
28.	Subjects found that having a job or doing an internship facilitates social inclusion and interaction	Moderate	Moderate concerns about methodological limitations and relevance	Kalef 2014 [57], Lewis 2013 [58], Mikkelsgard 2014 [61], Nieminen 2012 [62]
29.	Having an occupation allowed persons with chronic diseases to focus on something other than the disease	Low	Moderate concerns about methodological limitations, relevance and adequacy	Areberg 2013 [45], Mikkelsgard 2014 [61], Ramon 2011 [63]
30.	Participants found it very important to do something that they could enjoy	Very low	Serious concerns about methodological limitations, relevance and adequacy; moderate concerns about coherence	Boycott 2015 [46], Ramon 2011 [63]
31.	Having a job was associated with an increase in self-esteem and self-confidence	Low	Moderate concerns about methodological limitations and relevance; minor concerns regarding adequacy	Lewis 2013 [58], Mikkelsgard 2014 [61], Nieminen 2012 [62]

* Assessed using CERQual [34,40].

## Supplementary Materials

The following are available online at http://www.mdpi.com/1660-4601/15/5/1022/s1.

## Appendix A

### Characteristics of Included Studies

**Andersen, 2014 [44]**	
Context	Denmark; multidisciplinary, coordinated and early return-to-work (RTW) intervention under the management of the municipal sickness benefit offices; program aimed at employed persons on sick leave due to complex health-related problems irrespective of medical diagnoses; participation obligatory if the municipal sickness benefit officer estimates that it would enhance the chances of RTW
Participants	17 workers (13 women); average age 44 years (range 23–61); on sick leave for approximately 8 weeks due to common mental problems; at the last interview 6 were still sick-listed, 3 were no longer sick-listed but unemployed, 8 had returned to work
Methods	Semi-structured interviews at 3 time points: (1) before intervention; (2) approximately 3 months after first interview; (3) 6–7 months after first interview; methodology in line with principles of the Interpretative Phenomenological Analysis
**Areberg, 2013 [45]**	
Context	Sweden; individual placement and support to help people with severe mental illness to find regular employment; data collected 12 months after the first meeting between participants and employment specialist; ongoing support
Participants *	17 participants (7 female), 20–59 years old, with a diagnosis of schizophrenia, psychosis, affective disorder or other; 2 employed and working at the time of the interview, 2 in a position of repose, 4 in a practice place, 2 in education, 7 looking for a job
Methods	Semi-structured interviews 12 months after first contact with employment specialist; stratified sampling; content analysis
* Participants from the same RCT as Hasson [52] who published a qualitative study based on interviews performed in the first year of implementation.	
**Boycott, 2015 [46]**	
Context	UK; individual placement and support for unemployed persons with severe mental illness recruited from community mental health teams and early intervention in psychosis teams; service offered for at least 6 months
Participants	31 participants (9 female) of the program who received IPS for at least 6 months; mean age 30.8 years; 14 psychosis, 8 schizophrenia, 4 bipolar disorder, 4 depression, 1 other diagnosis; 12 participants had gained employment at interview
Methods	Semi-structured interviews; interview after at least six months receiving the intervention; purposive sampling; thematic analysis
**Buus, 2015 [47]**	
Context	Denmark; two counselling sessions by an occupational physician encouraging low-back pain (LBP) patients who expressed concerns about their ability to maintain their current job to engage in their own treatment by changing work routines and by exercising; a workplace visit was carried out if required
Participants	25 LBP patients (14 female); mean age 46.8 years
Methods	Semi-structured interviews with patients 1–4 years after concluding the counselling intervention; purpose sample; theoretical framework: “interactionism”; analysis: interpretative thematic approach
**Cameron, 2012 [48]**	
Context	UK, South of England; vocational guidance model that aims to help workers with mental health problems to retain their employment; project workers listen, clarify, give information, refer on to other services and collaborate to draw up client action plans; referrals to the project are done by general practitioners, other health workers, employers, or by clients themselves
Participants	14 participants (10 female), average age 43.5 years (range 29–54)
Methods	Semi-structured individual interviews with project users; critical realist methodology
**De Blasi, 2014 [49]**	
Context	France; support groups for vocational rehabilitation integrated in a multidisciplinary hospital department for patients who wish to RTW or who have already returned to work and have difficulties or questions regarding to work; led by an occupational therapist and a psychologist; 4 monthly sessions of 90 min
Participants	20 cancer patients (17 female); mean age at diagnosis: 47 years (range 37–55); cancer site: 16 breast, 2 hematopoietic, 1 digestive, 1 ear-nose-throat; 19 of them employed at diagnosis; 12 of them on sick leave during support group, 8 already working
Methods	Open questions in the questionnaire sent to participants one month after the last session; thematic analysis
**Germundsson, 2011 [50]**	
Context	Sweden; collaboration projects from a Swedish project group (NTG-New Routes to the Labour Market-Quality Assurance of Collaboration) within the Equal program; unemployed persons with mental health problems are part of the target population; duration: not found
Participants	(A) 8 service users (4 female), aged 19–52 years, unemployed and in vocational rehabilitation, sick-listed for 18–60 months due to mental health problems, six of them with little or no experience of paid employment; (B) 20 professionals (results for Group B not reported here)
Methods	Semi-structured interviews with service users on two occasions approximately six months apart; purposeful sample; thematic analysis guided by a theoretical framework
**Glavare, 2012 [51]**	
Context	Sweden; multi-professional pain-rehabilitation followed by a coached work-training (CWT) program; inclusion criteria for CWT were: unemployment, motivation for return-to work and at least 50% work capacity; the CWT program lasted 3–6 months
Participants	11 participants (8 female); 22–58 years-old; with long-term musculoskeletal pain, who had participated in the CWT program, 8 employed at interview
Methods	Semi-structured interviews; interviews performed 4–21 (mean = 11) months after the program; Grounded theory approach
**Hasson, 2011 [52]**	
Context	Sweden (Malmö, Southern Sweden); individual placement and support to help people with severe mental illness to find regular employment; ongoing support at work
Participants	15 clients of the service; characteristics not reported
Methods	Key informant interviews, non-participant observations and document analysis. Interviews with (A) 15 clients and (B) other actors (e.g., employment specialists, project head, mental health care staff, social insurance officers; results for Group B not considered in this review; content analysis
**Haugli, 2011 [53]**	
Context	Norway; multidisciplinary occupational rehabilitation program for patients on long-term sick leave, considered to have a fair chance of return-to work; it included different physical activities and individual and group based counselling aiming to increase function and work related processes; duration: not found
Participants	20 individuals with musculoskeletal and/or psychological complaints; 10 had returned to work (3 men 46–58 years old, 7 women 41–56 years old) and 10 individuals were registered with a disability pension (3 men 41–53 years old, 7 women 41–56 years old)
Methods	Semi-structured telephone interviews with individuals who attended the rehabilitation program 3 years ago; purposive sampling; systematic text condensation inspired by Giorgi’s phenomenological analysis
**Higgins, 2014 [54]**	
Context	UK, Northern Ireland; the role of the general practitioner when managing illness that may deserve long-term sick leave is investigated; duration: not applicable
Participants	(A) 9 employees who had experienced a long-term sickness absence and (B) other stakeholders (e.g., government-level policy makers, trust-level human resources managers, occupational health clinicians; results for Group B not presented here)
Methods	Comparative case study within three Health and Social Care Trusts; 61 semi-structured interviews and 3 focus group; purposive sampling for the Trusts, otherwise convenience sampling; theoretically framed by Talcott Parsons’s model of the medical contribution to the sick role; realistic approach
**Hubertsson, 2011 [55]**	
Context	Sweden; two actors in the standard management of sickness absence are investigated: the Social Insurance Agency and Health Care; duration: not applicable
Participants	15 participants (11 female); aged 33–63 years; with experience of sickness absence due to musculoskeletal disorders, 5 partly working, 2 partly unemployed, 5 on sick leave, 3 on disability pension
Methods	Semi-structured interviews; purposive sampling; latent content analysis
**Jansson, 2014 [56]**	
Context	Sweden, two counties; two strategies that aim to improve the work ability of persons with common mental disorders are investigated: (1) a pedagogical model of learning called problem-based method, lasting 12 weeks and (2) cognitive behavioral therapy, number of sessions not standardized
Participants	16 participants (14 female); aged 30 to 63 years; 8 people from each intervention and each county; 13 working, 3 in sick leave
Methods	Semi-structured interviews; purposive sampling; content analysis
**Kalef, 2014 [57]**	
Context	Norway; job training program at Norway’s largest telecommunications company with financial support from Norway’s Labour and Welfare Organization (NAV), targeted at people with disabilities with a high school diploma and motivated to gain employment; three main components: computer training, personal development, and work-practice; duration: not found
Participants	(A) 15 completers of program, 14 current participants, and (B) other stakeholders (e.g., company leaders, program administrators, teachers, community members; results for Group B not presented here)
Methods	Personal observations, 29 semi-structured interviews and document review; purposive sampling; thematic analysis
**Lewis, 2013 [58]**	
Context	UK; personalized tailored supported employment intervention to help people with disabilities to find employment; funded by the Department for Work and Pensions and managed by its agency, Jobcentre Plus; variable duration, depending on length of absence and follow-along supports
Participants	98 participants (40 female) from a sample of 11 providers; median age group 40–49 years, the primary disability of 48% of participants was a learning disability
Methods	Semi-structured interviews; purposive sampling; thematic analysis
**Martin, 2012 [60]**	
Context	Denmark; multidisciplinary, coordinated and tailored RTW-intervention for employees with relatively mild mental health problems *; sick-listed for 4–12 weeks; collaboration between a Danish municipality and a private company specialized in RTW; duration of intervention: 12 weeks
Participants	(A) 10 persons who completed the intervention; (B) other stakeholders (i.e., municipal social insurance officers and the multidisciplinary team); results for Group B not considered here
Methods	Interviews with participants were audio-taped, transcribed verbatim and coded thematically with the software NVivo
* Same intervention as in Martin (2015) [59] but implemented in only one municipality.	
**Martin, 2015 [59]**	
Context	Denmark; multidisciplinary, coordinated and tailored return-to-work-intervention for employees with common mental health problems; sick-listed for 4–12 weeks; collaboration between three Danish municipalities and a private company specialized in RTW; duration of intervention: 12 weeks
Participants	(A) 10 persons who completed the intervention, from three different municipalities; and (B) other stakeholders (i.e., municipal social insurance officers and the multidisciplinary team); results for Group B not considered here
Methods	Interviews with participants were audio-taped, transcribed verbatim and coded thematically with the software NVivo.
* Same intervention as in Martin (2012) [60] but implemented in 3 further municipalities.	
**Mikkelsgard, 2014 [61]**	
Context	Norway; supported employment project mainly targeted at people under the age of 25 years who receive financial assistance from the NAV (Norwegian Labour and Welfare Administration) and have mental health problems; collaboration between NAV and mental health services; voluntary participation based on individual motivation; duration: not found
Participants	Seven out of 52 participants of the project (5 women, 2 men); 20–35 years of age
Methods	Semi-structured interviews, audio-recorded and transcribed verbatim; content analysis
**Nieminen, 2012 [62]**	
Context	Eight European countries; lifelong learning strategy to facilitate social inclusion of unemployed persons with severe mental illness. Participants were offered training related to personal development and planning, employment and recovery, as well as opportunities for unpaid and paid activities *; duration: not found
Participants	23 service users (9 female, 11 male, 3 not known); mean age 42.8 years (range 24–57); with schizophrenia, schizoaffective or bipolar disorder
Methods	Interviews (2–5 key informants from each site), chosen at random and interviewed at baseline, and 10 and 20 months; thematic approach
* Same intervention as Ramon (2011) [63]; both publications seem to be based on the same interviews; only additional information to Ramon 2011 has been extracted.	
**Ramon, 2011 [63]**	
Context	(see Nieminen, 2012) *
Participants	Service users with schizophrenia, schizoaffective or bi-polar disorder
Methods	Semi-structured interviews (2–5 in each site) at baseline and 21 interviews 10 months later; 138 written self-reports at baseline and 99 ten months later
* Same intervention as Nieminen (2012) [62].	
**Reagon, 2011 [64]**	
Context	UK, Wales; condition management programs (CMPs) led by the National Health Service to help individuals to manage health conditions better in relation to a return-to-work; characterized by a biopsychosocial approach and cognitive behavioral therapy techniques; clients are voluntarily referred to the CMP by the Jobcentre; about 13 weeks intervention after the evaluation, depending on individual needs
Participants	(A) 15 participants (9 female) who had recently completed the program; 20–50 years-old; with musculoskeletal (*n* = 7) and mental health problems (*n* = 8) as primary health condition; (B) 12 staff members; results for Group B not considered in this review
Methods	Semi-structured interviews; tape-recorded and transcribed verbatim; purposive sample; thematic analysis
**Secker, 2012 [65]**	
Context	UK; Condition Management Programmes aimed at supporting persons on incapacity benefits due to long-term conditions to RTW; joint venture between the UK Department of Health and the Department for Work and Pensions; duration: not found
Participants	39 participants of the program (22 female) with mental health problems (*n* = 20), musculoskeletal (*n* = 8), cardiovascular (*n* = 4), and a combination of conditions (*n* = 7); most of them (*n* = 31) in their middle years
Methods	Twelve focus groups conducted at the site of the corresponding program; the discussions were tape-recorded and transcribed verbatim; purposive sample; text analysis
**Tiedtke, 2012 [66]**	
Context	Belgium; legislation affecting RTW of women with breast cancer; duration: not applicable
Participants	(A) 5 women who survived breast cancer and (B) 4 representatives of patient associations and further stakeholders (e.g., treating physicians, employers, social security physicians); results for Group B not considered in this review
Methods	Three multidisciplinary focus groups; discussions tape-recorded and transcribed verbatim; purposive sample; thematic analysis
**Vandekinderen, 2012 [67]**	
Context	Belgium; labor-market training program for women with mental health problems; five linear modules: registration, observation, guidance, training for employment, and work placement; duration: not found
Participants	(A) 11 women with mental health problems and substance dependence and (B) other stakeholders (i.e., the chief manager, support workers, and project partners); results for Group B not considered in this review
Methods	Document analysis of all available project documents and 17 interviews with women with mental health problems; the interviews were audio-taped, transcribed and returned to the participants for review; interpretative research approach
