# Supplementary material for: Views and Experiences of Persons with Chronic Diseases about Strategies that Aim to Integrate and Re-Integrate Them into Work: A Systematic Review of Qualitative Studies
Source: Int J Environ Res Public Health. 2018 May 18;15(5):1022. doi: 10.3390/ijerph15051022 (PMC5982061; doi:10.3390/ijerph15051022)
Supplement: Supplementary file 1 [file ijerph-15-01022-s001.pdf]

Supplementary Material 1

Views and experiences of persons with chronic diseases about strategies that aim to integrate and re-integrate them at work: a systematic review of qualitative studies

SEARCH STRATEGIES

Database: Medline
Interface: Ovid
Search date: April 2016

1	exp Disabled Persons/
2	(disabled or disabilit*).mp.
3	Chronic Disease/
4	(chronic ill* or chronic sick* or chronic absence* or chronic disease* or chronic condition*).mp.
5	(long standing sick* or long standing absence* or long standing disease* or long standing ill*).mp.
6	(longstanding sick* or longstanding absence* or longstanding disease* or longstanding ill*).mp.
7	llsi.ti,ab.
8	(long term sick* or long term absence* or long term disease* or long term ill*).mp.
9	(longterm sick* or longterm absence* or longterm disease* or longterm ill*).mp.
10	(permanent ill* or permanent absence* or permanent disease* or permanent sick*).mp.
11	Mental Disorders/
12	(mental illness* or mental disorder* or mental health issue* or mental diagnosis or mental diagnoses or mental health problem* or mental health disorder* or psychological illness* or psychiatric illness* or psychological disorder* or psychiatric disorder* or psych* condition* or psych* diagnoses or psych* diagnosis or psych* problem*).ti,ab,kf.
13	depression.ti.
14	Depressive Disorder/ or Depressive Disorder, Major/ or Depression, Postpartum/ or Dysthymic Disorder/
15	Nervous System Diseases/
16	(Neurologic* disorder* or neurologic* disease* or nervous system disorder* or nervous system disease*).ti,ab,kf.
17	Headache Disorders/ or exp Headache Disorders, Primary/
18	(Migraine* or headache*).ti,ab,kf.
19	Metabolic Disorders/ or Endocrine System Diseases/
20	(metabolic disorder* or metabolic disease* or endocrine disorder* or endocrine disease*).ti,ab,kf.
21	Diabetes Mellitus/ or Diabetes Mellitus, Type 1/ or Diabetes Mellitus, Type 2/

22	(diabetes or diabetic*).ab,kf,ti.
23	Musculoskeletal Diseases/
24	(musculoskeletal disorder* or musculoskeletal disease* or musculoskeletal complaint* or musculoskeletal condition* or musculoskeletal problem* or musculoskeletal diagnosis or musculoskeletal diagnoses).ab,kf,ti.
25	exp Back Pain/ or Neck Pain/
26	((neck or cervical) and (pain or ache)).ab,kf,ti.
27	((back or "spinal column") and (pain or ache)).ab,kf,ti.
28	(neckache or backache).ab,kf,ti.
29	Respiratory Tract Diseases/
30	(respiratory illness* or respiratory disease* or respiratory disorder* or respiratory condition* or respiratory tract illness* or respiratory tract disease* or respiratory tract disorder* or respiratory tract indication* or respiratory tract condition* or respiratory tract diagnosis or respiratory tract diagnoses).ab,kf,ti.
31	exp Pulmonary Disease, Chronic Obstructive/
32	(Chronic obstructive pulmonary disease or COPD or (bronch* and (disease* or disorder*) and chronic*) or pulmonary emphysema).ab,kf,ti.
33	Cardiovascular Diseases/
34	(cardiovascular disorder* or cardiovascular disease*).ab,kf,ti.
35	Myocardial Ischemia/ or Myocardial Infarction/
36	(ischemic heart disease or ischaemic heart disease or cardiac infarction or coronary infarction or heart attack or heart infarction). ab,kf,ti.
37	Neoplasms/
38	(cancer* or carcinoma* or neoplasm* or sarcoma* or tumor*).ab,kf,ti.
39	1 or 2 or 3 or 4 or 5 or 6 or 7 or 8 or 9 or 10 or 11 or 12 or 13 or 14 or 15 or 16 or 17 or 18 or 19 or 20 or 21 or 22 or 23 or 24 or 25 or 26 or 27 or 28 or 29 or 30 or 31 or 32 or 33 or 34 or 35 or 36 or 37 or 38
40	Employment/
41	exp Occupations/
42	Work/
43	Unemployment/
44	Occupation or occupations.mp.
45	work*.mp.
46	vocation*.mp.
47	(unemploy* or employ*).mp. or (labour or labor).ti,ab.
48	(job or jobs).mp.
49	(earn* or paid or paying or payment*).mp.
50	jobseek*.mp.
51	Income/ or "Salaries and Fringe Benefits"/
52	(salary or salari* or income or wages or waged or wage).mp.
53	40 or 41 or 42 or 43 or 44 or 45 or 46 or 47 or 48 or 49 or 50 or 51 or 52
54	rehabilitation/ or rehabilitation, vocational/
55	Education, Professional, Retraining/
56	welfare to work or welfare at work.mp.
57	(back to work or back into work).mp.
58	return to work.mp.

59	Training Support/
60	training.mp.
61	retraining.mp.
62	re-training.mp.
63	(skill or skills).mp.
64	advice.mp.
65	Counseling/
66	(counselling or counseling).mp.
67	Insurance, Disability/
68	disability benefit*.mp.
69	Social Security/
70	social security.mp.
71	Sick Leave/
72	sick leave.mp.
73	Retirement/
74	(mobility allowance* or mobility pension*).mp.
75	(disabilit* allowance* or disability* benefit*).mp.
76	sickness* benefit*.mp.
77	sickness* pension*or disability pension*.mp.
78	premature* retire*.mp.
79	early retire*.mp.
80	(quota or quotas).mp.
81	invalidity pension*.mp.
82	(invalidity allowance* or support allowance*).mp.
83	(invalidity benefit* or social benefit* or invalidity pension*).mp.
84	almp.ti,ab. or active labour market program*.mp. or active labor market program*.mp.
85	(employ* subsidy or employ* subsidies or wage subsidy or wage subsidies).mp.
86	(wage subsidy or wage subsidies).mp.
87	disability living allowance*.mp.
88	attendance allowance*.mp.
89	incapacity benefit*.mp.
90	incapacity allowance*.mp.
91	incapacity pension*.mp.
92	Employment, Supported/
93	supported work.mp.
94	supported employ*.mp.
95	(tax incentive* or tax allowance* or tax credit*).mp.
96	work preparation*.mp.
97	Case Management/
98	(Employment service* or case management).mp.
99	work focused interview*.mp.
100	(work trial or work trials).mp.
101	(employment trial or employment trials).mp.
102	(work placement* or job placement).mp.
103	(sheltered work* or sheltered employ*).mp.

104	(work preparation or job preparation).mp.
105	(workstep or work-step).mp.
106	(job match or workfare or work fare).mp.
107	access to work.mp.
108	jobmatch.mp.
109	(Worksite accommodation* or workplace accommodation* or work-place accommodation* or work accommodation* or job accommodation* or employment accommodation* or reasonable accommodation*).mp.
110	54 or 55 or 56 or 57 or 58 or 59 or 60 or 61 or 62 or 63 or 64 or 65 or 66 or 67 or 68 or 69 or 70 or 71 or 72 or 73 or 74 or 75 or 76 or 77 or 78 or 79 or 80 or 81 or 82 or 83 or 84 or 85 or 86 or 87 or 88 or 89 or 90 or 91 or 92 or 93 or 94 or 95 or 96 or 97 or 98 or 99 or 100 or 101 or 102 or 103 or 104 or 105 or 106 or 107 or 108 or 109
111	39 and 53 and 110
112	limit 111 to (abstracts and english language and yr="2011 - 2016")

Database: PSYCinfo
Interface: EBSCOhost
Search date: April 2016

1	DE "Multiple Disabilities" OR DE "Disabilities"
2	DE "Chronic Illness" OR DE "Chronicity (Disorders)"
3	DE "Physical Disorders" AND (TI chronic or KW chronic)
4	TI (disabled or disabilit*or "chronic sick*" or "chronic ill*" or "chronic absence*" or "chronic disease*" or "chronic condition*") OR AB (disabled or disability* or "chronic sick*" or "chronic ill*" or "chronic absence*" or "chronic disease*" or "chronic condition*") OR DE (disabled or disability* or "chronic sick*" or "chronic ill*" or "chronic absence*" or "chronic disease*" or "chronic condition*") OR KW (disabled or disability* or "chronic sick*" or "chronic ill*" or "chronic absence*" or "chronic disease*" or "chronic condition*")
5	TI ("long standing sick*" or "long standing absence*" or "long standing disease*" or "long standing ill*") OR AB ("long standing sick*" or "long standing absence*" or "long standing disease*" or "long standing ill*") OR DE ("long standing sick*" or "long standing absence*" or "long standing disease*" or "long standing ill*") OR KW ("long standing sick*" or "long standing absence*" or "long standing disease*" or "long standing ill*")
6	TI ("longstanding sick*" or "longstanding absence*" or "longstanding disease*" or "longstanding ill*") OR AB ("longstanding sick*" or "longstanding absence*" or "longstanding disease*" or "longstanding ill*") OR DE ("longstanding sick*" or "longstanding absence*" or "longstanding disease*" or "longstanding ill*") OR KW ("longstanding sick*" or "longstanding absence*" or "longstanding disease*" or "longstanding ill*")
7	TI ("long term sick*" or "long term absence*" or "long term disease*" or "long term ill*") OR AB ("long term sick*" or "long term absence*" or "long term disease*" or "long term ill*") OR DE ("long term sick*" or "long term absence*" or "long term disease*" or "long term ill*") OR KW ("long term sick*" or "long term absence*" or "long term disease*" or "long term ill*")
8	TI ("permanent absence*" or "permanent disease*" or "permanent sick*" or "permanent ill*") OR AB ("permanent absence*" or "permanent disease*" or "permanent sick*" or "permanent ill*") OR DE ("permanent absence*" or "permanent disease*" or "permanent sick*" or "permanent ill*") OR KW ("permanent absence*" or "permanent disease*" or "permanent sick*" or "permanent ill*")
9	TI llsi OR AB llsi
10	DE "Chronic Mental Illness"
11	DE "Mental Disorders"
12	TI ("mental illness*" or "mental disorder*" or "mental health issue*" or "mental diagnosis" or "mental diagnoses" or "mental health problem*" or "mental health disorder*" or "psychological illness*" or "psychiatric illness*" or "psychological disorder*" or " psychiatric disorder*" or "psych* condition*" or "psych* diagnoses" or "psych* diagnosis" or "psych* problem*") OR AB ("mental illness*" or "mental disorder*" or "mental health issue*" or "mental diagnosis" or "mental diagnoses" or "mental health problem*" or "mental health disorder*" or "psychological illness*" or "psychiatric illness*" or "psychological disorder*" or " psychiatric disorder*" or

	"psych* condition*" or "psych* diagnoses" or "psych* diagnosis" or "psych* problem*") OR KW ("mental illness*" or "mental disorder*" or "mental health issue*" or "mental diagnosis" or "mental diagnoses" or "mental health problem*" or "mental health disorder*" or "psychological illness*" or "psychiatric illness*" or "psychological disorder*" or " psychiatric disorder*" or "psych* condition*" or "psych* diagnoses" or "psych* diagnosis" or "psych* problem*")
13	DE "Major Depression" OR DE "Dysthymic Disorder" OR DE "Endogenous Depression" OR DE "Postpartum Depression" OR DE "Reactive Depression" OR DE "Recurrent Depression" OR TI depression
14	DE "Nervous System Disorders"
15	TI ("Neurologic* disorder*" or "neurologic* disease*" or "nervous system disorder*" or "nervous system disease*") OR AB ("Neurologic* disorder*" or "neurologic* disease*" or "nervous system disorder*" or "nervous system disease*") OR KW ("Neurologic* disorder*" or "neurologic* disease*" or "nervous system disorder*" or "nervous system disease*")
16	DE "Headache" OR DE "Migraine Headache" OR DE "Muscle Contraction Headache"
17	TI (migraine or headache) OR AB (migraine or headache) OR KW (migraine or headache)
18	DE "Metabolism Disorders" OR DE "Endocrine Disorders"
19	TI ("metabolic disorder*" or "metabolic disease*" or "endocrine disorder*" or "endocrine disease*") OR AB ("metabolic disorder*" or "metabolic disease*" or "endocrine disorder*" or "endocrine disease*") OR KW ("metabolic disorder*" or "metabolic disease*" or "endocrine disorder*" or "endocrine disease*")
20	DE "Diabetes" OR DE "Diabetes Mellitus" OR DE "Type 2 Diabetes"
21	TI (diabetes or diabetic*) OR AB (diabetes or diabetic*) OR KW (diabetes or diabetic*)
22	DE "Musculoskeletal Disorders"
23	TI ("musculoskeletal disorder*" or "musculoskeletal disease*" or "musculoskeletal complaint*" or "musculoskeletal condition*" or "musculoskeletal problem*" or "musculoskeletal diagnosis" or "musculoskeletal diagnoses") OR AB ("musculoskeletal disorder*" or "musculoskeletal disease*" or "musculoskeletal complaint*" or "musculoskeletal condition*" or "musculoskeletal problem*" or "musculoskeletal diagnosis" or "musculoskeletal diagnoses") OR KW ("musculoskeletal disorder*" or "musculoskeletal disease*" or "musculoskeletal complaint*" or "musculoskeletal condition*" or "musculoskeletal problem*" or "musculoskeletal diagnosis" or "musculoskeletal diagnoses")
24	DE "Back Pain"
25	TI ((neck or cervical) and (pain or ache)) OR AB ((neck or cervical) and (pain or ache)) OR KW ((neck or cervical) and (pain or ache))
26	TI ((back or "spinal column") and (pain or ache))
27	TI (neckache or backache)
28	DE "Respiratory Tract Disorders"
29	TI ("respiratory illness*" or "respiratory disease*" or "respiratory disorder*" or "respiratory condition*" or "respiratory tract illness*" or "respiratory tract disease*" or "respiratory tract disorder*" or "respiratory tract indication*" or "respiratory tract condition*" or "respiratory tract diagnosis" or "respiratory tract diagnoses") OR AB

	("respiratory illness*" or "respiratory disease*" or "respiratory disorder*" or "respiratory condition*" or "respiratory tract illness*" or "respiratory tract disease*" or "respiratory tract disorder*" or "respiratory tract indication*" or "respiratory tract condition*" or "respiratory tract diagnosis" or "respiratory tract diagnoses") OR KW ("respiratory illness*" or "respiratory disease*" or "respiratory disorder*" or "respiratory condition*" or "respiratory tract illness*" or "respiratory tract disease*" or "respiratory tract disorder*" or "respiratory tract indication*" or "respiratory tract condition*" or "respiratory tract diagnosis" or "respiratory tract diagnoses")
30	DE "Chronic Obstructive Pulmonary Disease" OR DE "Bronchial Disorders" OR DE "Pulmonary Emphysema"
31	TI ("Chronic obstructive pulmonary disease" or COPD or (bronch* and (disease* or disorder*) and chronic*) or "pulmonary emphysema") OR AB ("Chronic obstructive pulmonary disease" or COPD or (bronch* and (disease* or disorder*) and chronic*) or "pulmonary emphysema") OR KW ("Chronic obstructive pulmonary disease" or COPD or (bronch* and (disease* or disorder*) and chronic*) or "pulmonary emphysema")
32	DE "Cardiovascular disorders"
33	TI ("cardiovascular disorder*" or "cardiovascular disease*") or AB ("cardiovascular disorder*" or "cardiovascular disease*") OR KW ("cardiovascular disorder*" or "cardiovascular disease*")
34	DE "Myocardial Infarctions"
35	TI ("ischemic heart disease" or "ischaemic heart disease" or "cardiac infarction" or "coronary infarction" or "heart attack" or "heart infarction") OR AB ("ischemic heart disease" or "ischaemic heart disease" or "cardiac infarction" or "coronary infarction" or "heart attack" or "heart infarction") OR KW ("ischemic heart disease" or "ischaemic heart disease" or "cardiac infarction" or "coronary infarction" or "heart attack" or "heart infarction")
36	DE "Neoplasms"
37	TI (cancer* or carcinoma* or neoplasm* or sarcoma* or tumor*) OR AB (cancer* or carcinoma* or neoplasm* or sarcoma* or tumor*) OR DE (cancer* or carcinoma* or neoplasm* or sarcoma* or tumor*) OR KW (cancer* or carcinoma* or neoplasm* or sarcoma* or tumor*)
38	S1 OR S2 OR S3 OR S4 OR S5 OR S6 OR S7 OR S8 OR S9 OR S10 OR S11 OR S12 OR S13 OR S14 OR S15 OR S16 OR S17 OR S18 OR S19 OR S20 OR S21 OR S22 OR S23 OR S24 OR S25 OR S26 OR S27 OR S28 OR S29 OR S30 OR S31 OR S32 OR S33 OR S34 OR S35 OR S36 OR S37
39	DE "Employment Status" OR DE "Self-Employment" OR DE "Unemployment" OR DE "Employability"
40	DE "Occupations" OR DE "Nontraditional Careers" OR DE "Occupational Status"
41	DE "Income Level" OR DE "Lower Income Level" OR DE "Middle Income Level" OR DE "Upper Income Level"
42	DE "Salaries"
43	TI (occupation or occupations or work* or vocation* or job or jobs or jobseek* or earn* or paid or paying or payment*) OR AB (occupation or occupations or work* or vocation* or job or jobs or jobseek* or earn* or paid or paying or payment*) OR KW (occupation or occupations or work* or vocation* or job or jobs or jobseek* or earn* or paid or paying or payment*)

44	TI (salary or salaries or income or wages or waged or wage or unemploy* or employ*) OR AB (salary or salaries or income or wages or waged or wage or unemploy* or employ*) OR KW (salary or salaries or income or wages or waged or wage or unemploy* or employ*)
45	TI (labour or labor) or AB (labour or labor) OR KW (labour or labor)
46	S39 OR S40 OR S41 OR S42 OR S43 OR S44 OR S45
47	DE "Rehabilitation" OR DE "Cognitive Rehabilitation" OR DE "Criminal Rehabilitation" OR DE "Drug Rehabilitation" OR DE "Neuropsychological Rehabilitation" OR DE "Neurorehabilitation" OR DE "Occupational Therapy" OR DE "Physical Therapy" OR DE "Psychosocial Rehabilitation"
48	DE "Vocational rehabilitation" OR DE "Supported employment" OR "Vocational evaluation" OR DE "Work adjustment training" OR DE "Disability management" OR DE "Rehabilitation counseling"
49	DE "On the Job Training"
50	DE "Disability Laws"
51	DE "Vocational Education" OR DE "Cooperative Education"
52	DE "Retirement" OR DE "Reemployment"
53	DE "Counseling"
54	DE "Social Security" OR DE "Disability Evaluation"
55	DE "Employee Leave Benefits"
56	TI ("welfare to work" or "welfare at work") OR AB ("welfare to work" or "welfare at work") OR DE ("welfare to work" or "welfare at work") OR KW ("welfare to work" or "welfare at work")
57	TI ("back to work" or "back into work") OR AB ("back to work" or "back into work") OR DE ("back to work" or "back into work") OR KW ("back to work" or "back into work")
58	TI ("sick leave" or "disability benefit*" or "disability pension*" or training or retraining or re-training or skill or skills or advice or counselling or counseling) OR AB ("sick leave" or "disability benefit*" or "disability pension*" or training or retraining or re-training or skill or skills or advice or counselling or counseling) OR DE ("sick leave" or disability benefit* or disability pension* or training or retraining or re-training or skill or skills or advice or counselling or counseling) OR KW ("sick leave" or "disability benefit*" or "disability pension*" or training or retraining or re-training or skill or skills or advice or counselling or counseling)
59	TI (quota or quotas or "mobility pension*" or "invalidity pension*" or "invalidity allowance*" or "invalidity benefit*" or "social benefit*" or "support allowance*") OR AB (quota or quotas or "mobility pension*" or "invalidity pension*" or "invalidity allowance*" or "invalidity benefit*" or "social benefit*" or "support allowance*") OR DE (quota or quotas or "mobility pension*" or "invalidity pension*" or "invalidity allowance*" or "invalidity benefit*" or "social benefit*" or "support allowance*") OR KW (quota or quotas or "mobility pension*" or "invalidity pension*" or "invalidity allowance*" or "invalidity benefit*" or "social benefit*" or "support allowance*")
60	TI ("disability living allowance*" or "attendance allowance*" or "incapacity benefit*" or "incapacity allowance*" or "incapacity pension*") OR AB ("disability living allowance*" or "attendance allowance*" or "incapacity benefit*" or "incapacity allowance*" or "incapacity pension*") OR DE ("disability living allowance*" or "attendance allowance*" or "incapacity benefit*" or "incapacity allowance*" or

	"incapacity pension*") OR KW ("disability living allowance*" or "attendance allowance*" or "incapacity benefit*" or "incapacity allowance*" or "incapacity pension*")
61	TI ("employ* subsidy" or "employ* subsidies" or "wage subsidy" or "wage subsidies" or "tax incentive*" or "tax allowance*" or "tax credit*" or "social security") OR AB ("employ* subsidy" or "employ* subsidies" or "wage subsidy" or "wage subsidies" or "tax incentive*" or "tax allowance*" or "tax credit*" or "social security") OR DE ("employ* subsidy" or "employ* subsidies" or "wage subsidy" or "wage subsidies" or "tax incentive*" or "tax allowance*" or "tax credit*" or "social security") OR KW ("employ* subsidy" or "employ* subsidies" or "wage subsidy" or "wage subsidies" or "tax incentive*" or "tax allowance*" or "tax credit*" or "social security")
62	TI (almp) OR AB (almp) OR TI ("active labour market program*" or "active labor market program*") OR AB ("active labour market program*" or "active labor market program*") OR DE ("active labour market program*" or "active labor market program*") OR KW ("active labour market program*" or "active labor market program*")
63	TI ("supported work or supported employ*") OR AB ("supported work or supported employ*") OR DE ("supported work or supported employ*") OR KW ("supported work or supported employ*")
64	TI ("work placement*" or "job placement*" or "employment service*" or "case management" or "work focused interview*") OR AB ("work placement*" or "job placement*" or "employment service*" or "case management" or "work focused interview*") OR DE ("work placement*" or "job placement*" or "employment service*" or "case management" or "work focused interview*") OR KW ("work placement*" or "job placement*" or "employment service*" or "case management" or "work focused interview*")
65	TI ("work preparation*" or "job preparation*" or "employment trial" or "employment trials" or "work trial" or "work trials" or workstep or work-step) OR AB ("work preparation*" or "job preparation*" or "employment trial" or "employment trials" or "work trial" or "work trials" or workstep or work-step) OR DE ("work preparation*" or "job preparation*" or "employment trial" or "employment trials" or "work trial" or "work trials" or workstep or work-step) OR KW ("work preparation*" or "job preparation*" or "employment trial" or "employment trials" or "work trial" or "work trials" or workstep or work-step)
66	TI ("job match" or workfare or "work fare" or "access to work") OR AB ("job match" or workfare or "work fare" or "access to work") OR DE ("job match" or workfare or "work fare" or "access to work") OR KW ("job match" or workfare or "work fare" or "access to work")
67	TI ("sheltered work*" or "sheltered employ*") OR AB ("sheltered work*" or "sheltered employ*") OR DE ("sheltered work*" or "sheltered employ*") OR KW ("sheltered work*" or "sheltered employ*")
68	TI ("worksite accommodation*" or "workplace accommodation*" or "work-place accommodation*" or "work accommodation*" or "job accommodation*" or "employment accommodation*" or "reasonable accommodation*") OR AB ("worksite accommodation*" or "workplace accommodation*" or "work-place accommodation*" or "work accommodation*" or "job accommodation*" or "employment accommodation*" or "reasonable accommodation*") OR DE

	("worksite accommodation*" or "workplace accommodation*" or "work-place accommodation*" or "work accommodation*" or "job accommodation*" or "employment accommodation*" or "reasonable accommodation*") OR KW ("worksite accommodation*" or "workplace accommodation*" or "work-place accommodation*" or "work accommodation*" or "job accommodation*" or "employment accommodation*" or "reasonable accommodation*")
69	DE "Accommodation (Disabilities)"
70	TI (Strategy or strategies or intervention* or program or programs or programme or programmes)
71	S47 OR S48 OR S49 OR S50 OR S51 OR S52 OR S53 OR S54 OR S55 OR S56 OR S57 OR S58 OR S59 OR S60 OR S61 OR S62 OR S63 OR S64 OR S65 OR S66 OR S67 OR S68 OR S69 OR 70
72	S38 AND S46 AND S71 (Limits: English, peer-reviewed, 2011-2016)

Databases: Cochrane Database of Systematic Reviews (CDSR), CRD-Database of Abstracts of Reviews of Effects (DARE) and CRD-Health Technology Assessment (HTA)
Interface: Ovid
Search date: April 2016

1	MeSH descriptor: [Disabled Persons] explode all trees
2	MeSH descriptor: [Chronic Disease] this term only
3	disabled or disabilit* or (chronic next sick*) or (chronic next ill*) or (chronic next absence*) or (chronic next disease*):ti,ab,kw (Word variations have been searched)
4	(long next standing next ill*) or (long next standing next sick*) or (long next standing next absence*) or (long next standing next disease*):ti,ab,kw (Word variations have been searched)
5	(longstanding next sick*) or (longstanding next ill*) or (longstanding next absence*) or (longstanding next disease*) or llsi:ti,ab,kw (Word variations have been searched)
6	(long next term next sick*) or (long next term next absence*) or (long next term next disease*):ti,ab,kw (Word variations have been searched)
7	(long next term next ill*) or (longterm next ill*) or (longterm next sick*) or (longterm next absence*) or (longterm next disease*):ti,ab,kw (Word variations have been searched)
8	(permanent next sick*) or (permanent next ill*) or (permanent next absence*) or (permanent next disease*):ti,ab,kw (Word variations have been searched)
9	(#1 or #2 or #3 or #4 or #5 or #6 or #7 or #8)
10	MeSH descriptor: [Employment] this term only
11	MeSH descriptor: [Occupations] explode all trees
12	MeSH descriptor: [Work] this term only
13	MeSH descriptor: [Unemployment] explode all trees
14	MeSH descriptor: [Income] this term only
15	MeSH descriptor: [Salaries and Fringe Benefits] this term only
16	(Occupation or occupations or work* or vocation* or job or jobs or earn* or paid or paying or payment*):ti,ab,kw (Word variations have been searched)
17	(salary or salari* or income or wages or waged or wage or unemploy* or employ*

	or labour or labor):ti,ab,kw (Word variations have been searched)
18	(#10 or #11 or #12 or #13 or #14 or #15 or #16 or #17)
19	MeSH descriptor: [Rehabilitation] this term only
20	MeSH descriptor: [Rehabilitation, Vocational] this term only
21	MeSH descriptor: [Education, Professional, Retraining] this term only
22	MeSH descriptor: [Training Support] this term only
23	MeSH descriptor: [Counseling] this term only
24	MeSH descriptor: [Insurance, Disability] this term only
25	MeSH descriptor: [Insurance, Liability] this term only
26	MeSH descriptor: [Social Security] explode all trees
27	MeSH descriptor: [Sick Leave] this term only
28	MeSH descriptor: [Retirement] this term only
29	MeSH descriptor: [Employment, Supported] this term only
30	"welfare to work" or "welfare at work" or "back to work" or "back into work":ti,ab,kw (Word variations have been searched)
31	(sick next leave) or (disability next benefit*) or (disability next pension*) or training or retraining or (re next training) or skill or skills or advice or counselling or counseling:ti,ab,kw (Word variations have been searched)
32	(mobility next allowance*) or (disabilit* next allowance*) or (sickness* next benefit*) or (sickness* next pension*) or (premature* next retire*) or (early next retire*):ti,ab,kw (Word variations have been searched)
33	(quota or (mobility next pension*) or (invalidity next pension*) or (invalidity next allowance*) or (invalidity next benefit*) or quotas or (employ* next subsidy) or (employ* next subsidies)):ti,ab,kw (Word variations have been searched)
34	(disability next living next allowance*) or (attendance next allowance*) or (incapacity next benefit*) or (incapacity next allowance*) or (incapacity next pension*) or (severe next disablement next allowance*):ti,ab,kw (Word variations have been searched)
35	(supported next work) or (supported next employ*) or (disabled next persons next tax next allowance*) or (access next to next work next program*):ti,ab,kw (Word variations have been searched)
36	((work next preparation*) or (work next focussed next interview*) or (work next focused next interview*) or workstep or (work next step)):ti,ab,kw (Word variations have been searched)
37	(disability next working next allowance*) or (condition next management next program*) or "work trial" or "work trials":ti,ab,kw (Word variations have been searched)
38	"employment trial" or "employment trials" or (work next placement*) or (disability next discrimination next act) or (work next preparation):ti,ab,kw (Word variations have been searched)
39	(job next preparation) or (work next preparation) or (job next placement) or (work next placement) or (job next match) or (employment next service*) or (case next management) or workfare or (work next fare):ti,ab,kw (Word variations have been searched)
40	"access to work" or jobmatch or (active next labour next market next program*) or (active next labor next market next program*) or almp or (worksite next accommodation*) or (workplace next accommodation*) or (work-place next

	accommodation*) or (work next accommodation*) or (job next accommodation*) or (employment next accommodation*) or (reasonable next accommodation*):ti,ab,kw (Word variations have been searched)
41	(#19 or #20 or #21 or #22 or #23 or #24 or #25 or #26 or #27 or #28 or #29 or #30 or #31 or #32 or #33 or #34 or #35 or #36 or #37 or #38 or #39 or #40)
42	(#9 and #18 and #41)
43	MeSH descriptor: [Mental Disorders] this term only
44	(mental illness*) or (mental disorder*) or (mental health issue*) or (mental diagnosis) or (mental diagnoses) or (mental health problem*) or (mental health disorder*) or (psychological illness*) or (psychiatric illness*) or (psychological disorder*) or (psychiatric disorder*) or (psych* condition*) or (psych* diagnoses) or (psych* diagnosis) or (psych* problem*):ti,ab,kw (Word variations have been searched)
45	depression:ti (Word variations have been searched)
46	MeSH descriptor: [Depressive Disorder] this term only
47	MeSH descriptor: [Depressive Disorder, Major] this term only
48	MeSH descriptor: [Nervous System Diseases] this term only
49	(Neurologic* disorder*) or (neurologic* disease*) or (nervous system disorder*) or (nervous system disease*):ti,ab,kw (Word variations have been searched)
50	MeSH descriptor: [Headache Disorders] this term only
51	MeSH descriptor: [Headache Disorders, Primary] explode all trees
52	(Migraine*) or (headache*):ti,ab,kw (Word variations have been searched)
53	MeSH descriptor: [Metabolic Diseases] this term only
54	MeSH descriptor: [Endocrine System Diseases] this term only
55	(metabolic disorder) * or (metabolic disease*) or (endocrine disorder*) or (endocrine disease*):ti,ab,kw (Word variations have been searched)
56	MeSH descriptor: [Diabetes Mellitus] this term only
57	MeSH descriptor: [Diabetes Mellitus, Type 1] this term only
58	MeSH descriptor: [Diabetes Mellitus, Type 2] this term only
59	(diabetes) or (diabetic*):ti,ab,kw (Word variations have been searched)
60	MeSH descriptor: [Musculoskeletal Diseases] this term only
61	(musculoskeletal disorder*) or (musculoskeletal disease*) or (musculoskeletal complaint*) or (musculoskeletal condition*) or (musculoskeletal problem*) or (musculoskeletal diagnosis) or (musculoskeletal diagnoses):ti,ab,kw (Word variations have been searched)
62	MeSH descriptor: [Neck Pain] this term only
63	MeSH descriptor: [Back Pain] explode all trees
64	(neck or cervical) and (pain or ache):ti,ab,kw (Word variations have been searched)
65	(back or "spinal column") and (pain or ache):ti,ab,kw (Word variations have been searched)
66	(neckache) or (backache):ti,ab,kw (Word variations have been searched)
67	MeSH descriptor: [Respiratory Tract Diseases] this term only
68	(respiratory illness*) or (respiratory disease*) or (respiratory disorder*) or (respiratory condition*) or (respiratory tract illness*) or (respiratory tract disease*) or (respiratory tract disorder*) or (respiratory tract indication*) or (respiratory tract condition*) or (respiratory tract diagnosis) or (respiratory tract diagnoses):ti,ab,kw (Word variations have been searched)

69	MeSH descriptor: [Pulmonary Disease, Chronic Obstructive] explode all trees
70	(Chronic obstructive pulmonary disease) or COPD or (bronch* next (disease* or disorder*) next chronic*) or (pulmonary emphysema):ti,ab,kw (Word variations have been searched)
71	MeSH descriptor: [Cardiovascular Diseases] this term only
72	(cardiovascular disorder*) or (cardiovascular disease*):ti,ab,kw (Word variations have been searched)
73	MeSH descriptor: [Myocardial Ischemia] explode all trees
74	(ischemic heart disease) or (ischaemic heart disease) or (cardiac infarction) or (coronary infarction) or (heart attack) or (heart infarction):ti,ab,kw (Word variations have been searched)
75	MeSH descriptor: [Neoplasms] this term only
76	(cancer*) or (carcinoma*) or (neoplasm*) or (sarcoma*) or (tumor*):ti,ab,kw (Word variations have been searched)
77	(#43 or #44 or #45 or #46 or #47 or #48 or #49 or #50 or #51 or #52 or #53 or #54 or #55 or #56 or #57 or #58 or #59 or #60 or #61 or #62 or #63 or #64 or #65 or #66 or #67 or #68 or #69 or #70 or #71 or #72 or #73 or #74 or #75 or #76)
78	(#9 or #77)
79	(#78 and #18 and #41)<br>Online Publication Date from Jan 2011 to Apr 2016, in Cochrane Reviews (Reviews only)
81	(#78 and #18 and #41)<br>Publication Year from 2011 to 2016, in Other Reviews
82	(#78 and #18 and #41)<br>Publication Year from 2011 to 2016, in Health Technology Assessment

Supplementary Material 2

Views and experiences of persons with chronic diseases about strategies that aim to integrate and re-integrate them at work: a systematic review of qualitative studies

CERQual evidence profile

(based on the methodology presented in Lewin et al. 2015 and Ames et al. 2017)

Table 1: CERQual evidence profile for the findings of the review

USE OF INDIVIDUAL AND HOLISTIC APPROACH
**Finding 1:** Persons with chronic health conditions wanted professionals to show a genuine interest

to understand and accept them as individuals	
**Assessment for each CERQual component**	
Methodological limitations	Moderate concerns regarding methodological limitations due to poor reporting of sampling in six studies, data collection in three studies and reflexivity in five studies
Coherence	No or minor concerns regarding coherence
Relevance	Moderate concerns due to a narrow range of settings (seven studies in Nordic countries and one study in UK; most participants with MD; different strategies)
Adequacy	No or minor concerns regarding adequacy
**Overall CERQual assessment**	
Moderate confidence	Due to moderate concerns about methodological limitations and moderate concerns about relevance
**Contributing studies**	Andersen 2014; Areberg 2013; Glavare 2012; Haugli 2011; Hubertsson 2011; Martin 2012; Mikkelsgard 2014; Reagon 2011
**Finding 2:** Persons with chronic conditions found it helpful to be seen from different perspectives (e.g. medical, psychological) and in more areas than the one directly related to work	
**Assessment for each CERQual component**	
Methodological limitations	Moderate concerns due to poor reporting of sampling in four studies, data collection in three studies and reflexivity in five studies
Coherence	No or very minor concerns regarding coherence
Relevance	Moderate concerns regarding relevance due to a narrow range of settings (five studies in Nordic countries, two in UK, one in Belgium; most participants with MHD and MSD)
Adequacy	No or minor concerns regarding adequacy
**Overall CERQual assessment**	
Moderate confidence	Due to moderate concerns regarding methodological limitations and moderate concerns about relevance
**Contributing studies**	Andersen 2014; Glavare 2012; Lewis 2013; Martin 2012; Martin 2015; Mikkelsgard 2014; Secker 2012; Vandekinderen 2012
**Finding 3:** Flexibility in the implementation of the strategy and the contact with the professionals was highly appreciated by the participants	

**Assessment for each CERQual component**	
Methodological limitations	Moderate concerns due to poor reporting of sampling in seven studies, reflexivity in eight studies
Coherence	No or very minor concerns regarding coherence
Relevance	Moderate concerns regarding relevance due to a narrow range of settings (seven studies in Nordic countries, four in UK, one in Belgium; most participants with MHD and MSD; most unemployed)
Adequacy	No or minor concerns regarding adequacy
**Overall CERQual assessment**	
Moderate confidence	Due to moderate concerns about methodological limitations and moderate concerns about relevance
**Contributing studies**	Andersen 2014; Areberg 2013; Cameron 2012; Germundsson 2011; Glavare 2012; Kalef 2014; Lewis 2013; Martin 2012; Mikkelsgard 2014; Reagon 2011; Secker 2012; Vandekinderen 2012
**CLARITY OF THE INTEGRATION STRATEGY AND PROCESS**	
**Finding 4:** Providing a definition and clarification of the problem and setting a clear course of action was found helpful	
**Assessment for each CERQual component**	
Methodological limitations	Minor concerns due to few information about non-participants, data collection and analysis and no discussion on reflexivity in one study
Coherence	No or minor concerns regarding coherence
Relevance	Moderate concerns regarding relevance due to narrow range of settings (one study in Denmark, one study in UK)
Adequacy	Moderate concerns due to the quantity of data
**Overall CERQual assessment**	
Low confidence	Due to minor concerns about methodological limitations and moderate concerns about relevance and adequacy
**Contributing studies**	Andersen 2014; Secker 2012
**Finding 5:** The role of activities and professionals is sometimes difficult to understand, leading to confusion and demotivation	

**Assessment for each CERQual component**	
Methodological limitations	Moderate concerns due to poor reporting on sampling and data collection in two studies, and lack of reporting or poor discussion of reflexivity in three studies
Coherence	No or minor concerns regarding coherence
Relevance	Moderate concerns due to narrow range of settings (four studies in Nordic countries, all participants suffering from MHD)
Adequacy	Minor concerns due to the quantity of data
**Overall CERQual assessment**	
Moderate confidence	Due to moderate concerns about methodological limitations and relevance
**Contributing studies**	Andersen, 2014; Germundsson 2011; Martin, 2012; Mikkelsgard, 2014
**Finding 6:** Some persons found it difficult to move forward because of receiving wrong or insufficient information about requisites, procedures and decisions affecting their professional integration	
**Assessment for each CERQual component**	
Methodological limitations	Moderate concerns due to poor reporting of setting and data collection and poor discussion of reflexivity
Coherence	No or minor concerns regarding coherence
Relevance	Moderate concerns due to only three studies (Belgium, Norway, Sweden)
Adequacy	Moderate concerns regarding adequacy due to quantity of data
**Overall CERQual assessment**	
Moderate confidence	Due to moderate concerns about methodological limitations, relevance and adequacy
**Contributing studies**	Hubertsson 2011; Mikkelsgard 2014; Tiedtke 2012
**Finding 7:** Collaboration between different professionals and agencies has positive consequences for the participants	
**Assessment for each CERQual component**	
Methodological limitations	Moderate concerns regarding poor information about participants and nonparticipants in three studies,

	reflexivity in two studies and data collection and analysis in one study
Coherence	No or minor concerns regarding coherence
Relevance	Substantial concerns regarding relevance due to narrow range of settings (two studies in Sweden, one study in Norway; all participants suffering from MHD)
Adequacy	Moderate concerns regarding adequacy due to quantity of data
**Overall CERQual assessment**	
Low confidence	Due to moderate concerns on methodological limitations and adequacy and substantial concerns on relevance
**Contributing studies**	Areberg 2013; Germundsson 2011; Mikkelsgard 2014
**TIMING OF THE REHABILITATION PROCESS**	
**Finding 8:** Some people would have liked to start working for their professional integration at an earlier stage	
**Assessment for each CERQual component**	
Methodological limitations	Moderate concerns due to poor reporting on setting, sampling, data collection and reflexivity
Coherence	No or minor concerns about coherence
Relevance	Minor concerns about relevance due to studies conducted in three countries and almost all participants employed
Adequacy	No or minor concerns about adequacy
**Overall CERQual assessment**	
Moderate confidence	Due to moderate concerns about methodological limitations and minor concerns about relevance
**Contributing studies**	Hubertsson 2011, Jansson 2014, Martin 2012, Martin 2015, Tiedtke 2012
**Finding 9:** A continuous rehabilitation process is positively experienced by subjects	
**Assessment for each CERQual component**	
Methodological limitations	Moderate concerns due to poor reporting on sampling, setting and data collection in one paper and on reflexivity in three studies

Coherence	No or minor concerns regarding coherence
Relevance	Moderate concerns regarding relevance due to a narrow range of settings (three studies in two Nordic countries, Sweden and Norway, participants with MHD and MSD)
Adequacy	Moderate concerns regarding adequacy due to quantity of data
**Overall CERQual assessment**	
Low confidence	Due to moderate concerns about methodological limitations, relevance and adequacy
**Contributing studies**	Germundsson 2011, Hubertsson 2011, Mikkelsgard 2014
**EXPERIENCE OF PERSONS WITH CHRONIC DISEASES WITH THE PROFESSIONALS**	
**Finding 10:** Perceiving professionals as experts increased the engagement of the participants with the intervention	
**Assessment for each CERQual component**	
Methodological limitations	Moderate concerns due to general poor reporting of sampling, and data collection and reflexivity in two studies
Coherence	No or minor concerns regarding coherence
Relevance	Minor concerns regarding relevance (six studies in Nordic and middle-European countries; different strategies and settings; participants in four studies with MHD)
Adequacy	No or minor concerns regarding adequacy
**Overall CERQual assessment**	
Moderate confidence	Due to moderate concerns about methodological limitations and minor concerns about relevance
**Contributing studies**	Andersen 2014, Areberg 2013, Cameron 2012, Lewis 2013, Martin 2015, Tiedtke 2012
**Finding 11:** The interaction style of the professional was perceived as a key element of the intervention	
**Assessment for each CERQual component**	
Methodological limitations	Moderate concerns due to poor reporting on sampling method, data collection and reflexivity
Coherence	No or minor concerns regarding coherence

Relevance	Minor concerns regarding relevance (nine studies in Nordic countries, UK and Belgium; different disease groups)
Adequacy	No or minor concerns regarding adequacy
**Overall CERQual assessment**	
Moderate confidence	Due to moderate concerns about methodological limitations and minor concerns about relevance
**Contributing studies**	Andersen 2014, Cameron 2012, Glavare 2012, Hubertsson 2011, Martin 2012, Mikkelsgard 2014, Reagon 2011, Secker 2012, Tiedtke 2012
**Finding 12:** Participants appreciated receiving practical support	
**Assessment for each CERQual component**	
Methodological limitations	Moderate concerns regarding representativeness of the study sample, and poor reporting of the context of data collection and reflexivity
Coherence	No or minor concerns regarding coherence
Relevance	Minor concerns regarding relevance (five studies in Nordic countries, five studies in UK and one in Belgium, different strategies and different long-term conditions)
Adequacy	No or minor concerns regarding adequacy
**Overall CERQual assessment**	
Moderate confidence	Due to moderate concerns about methodological limitations and minor concerns about relevance
**Contributing studies**	Areberg 2013, Boycott 2015, Cameron 2012, Glavare 2012, Higgins 2014, Kalef 2014, Lewis 2013, Martin 2015, Mikkelsgard 2014, Secker 2012, Tiedtke 2012
**Finding 13:** Stability and availability of the support person was associated with a sense of security and relief	
**Assessment for each CERQual component**	
Methodological limitations	Moderate concerns about poor reporting of representativeness of the sample and reflexivity
Coherence	No or minor concerns regarding coherence
Relevance	Moderate concerns due to a narrow range of settings (two studies in UK, one study in Norway)

Adequacy	No or minor concerns regarding adequacy
**Overall CERQual assessment**	
Moderate confidence	Due to moderate concerns about methodological limitations and substantial concerns about relevance
**Contributing studies**	Lewis 2013, Mikkelsgard 2014, Secker 2012
**Finding 14:** Professionals were a source of emotional support for some participants	
**Assessment for each CERQual component**	
Methodological limitations	Minor concerns due to poor reporting of representativeness of the sample, data collection and reflexivity
Coherence	No or minor concerns regarding coherence
Relevance	Moderate concerns regarding relevance due to a narrow range of settings (two studies in Nordic countries and two studies in UK; participants with MD in three studies)
Adequacy	No or minor concerns regarding adequacy
**Overall CERQual assessment**	
Moderate confidence	Due to minor concerns about methodological limitations and moderate concerns about relevance
**Contributing studies**	Areberg 2013, Boycott 2015, Cameron, 2012, Kalef, 2014
**CHANGES IN THE UNDERSTANDING OF HEALTH AND WORK SITUATION**	
**Finding 15:** Participants increased their understanding of symptoms, mechanisms of the disease and their health status	
**Assessment for each CERQual component**	
Methodological limitations	Minor concerns about poor reporting of representativeness of the sample (five studies minor concerns, two studies substantial concerns)
Coherence	No or minor concerns regarding coherence
Relevance	Minor concerns about relevance (four studies in Nordic countries, two in UK, one in France; different strategies; different long-term conditions)
Adequacy	Minor concerns regarding adequacy due to thin data in three studies

**Overall CERQual assessment**	
High confidence	Due to minor concerns about methodological limitations, coherence and adequacy
**Contributing studies**	Andersen 2014, Areberg 2013, Buus 2015, De Blasi 2014, Jansson 2014, Reagon 2011, Secker 2012
**Finding 16:** Professionals' focus on workplace conditions helped participants to broaden their understanding of work dynamics and showed them possible ways to be a resource for the company	
**Assessment for each CERQual component**	
Methodological limitations	Moderate concerns about poor reporting of data collection and poor discussion of reflexivity in two studies
Coherence	No or minor concerns regarding coherence
Relevance	Moderate concerns regarding relevance (three studies in three countries and two European regions; different strategies; different long-term conditions)
Adequacy	Minor concerns regarding adequacy (thin data)
**Overall CERQual assessment**	
Moderate confidence	Due to moderate concerns about methodological limitations and relevance and minor concerns regarding adequacy
**Contributing studies**	Buus 2015, Cameron 2012, Kalef 2014
**Finding 17:** Participants appreciated increasing their own understanding	
**Assessment for each CERQual component**	
Methodological limitations	Moderate concerns about representativeness of the sample, data collection and reflexivity
Coherence	No or minor concerns regarding coherence
Relevance	Moderate concerns regarding limited settings (two studies in Norway, one in UK and one study in different European countries; different strategies; most participants with MD)
Adequacy	No or minor concerns regarding adequacy
**Overall CERQual assessment**	
Moderate confidence	Due to moderate concerns about methodological limitations and relevance
**Contributing studies**	Cameron 2012, Haugli 2011, Kalef 2014, Nieminen 2012

ACTIVE INVOLVEMENT OF THE PERSONS WITH CHRONIC HEALTH CONDITIONS IN PROCESS OF PROFESSIONAL INTEGRATION	
**Finding 18:** Participants appreciated working actively and being involved in the development and implementation of the activities to improve their professional integration	
**Assessment for each CERQual component**	
Methodological limitations	Minor concerns due to poor reporting of representativeness of sample and discussion of reflexivity
Coherence	No or minor concerns regarding coherence
Relevance	Moderate concerns about relevance due five studies performed in Nordic countries (three in Sweden, one in Denmark, one in Norway) and one in UK; different diseases and strategies
Adequacy	No or minor concerns regarding adequacy
**Overall CERQual assessment**	
Moderate confidence	Due to minor concerns about methodological limitations and moderate concerns about relevance
**Contributing studies**	Areberg 2013, Buus 2015, Glavare 2012, Jansson 2014, Mikkelsgard 2014, Secker 2012
**COMPETENCIES DEVELOPED BY THE PARTICIPANT**	
**Finding 19:** Participants welcomed being taught skills to manage problematic situations in different areas of life	
**Assessment for each CERQual component**	
Methodological limitations	Moderate concerns due to poor reporting of representativeness of the sample, data collection and discussion of reflexivity
Coherence	Minor or no concerns regarding coherence
Relevance	Moderate concerns about relevance (five studies in three Nordic countries, four studies in UK, one study in different European countries; persons with MD in eight of 10 studies)
Adequacy	Minor o no concerns regarding adequacy
**Overall CERQual assessment**	
Moderate confidence	Due to moderate concerns about methodological limitations and relevance

**Contributing studies**	Boycott 2015, Cameron 2012, Glavare 2012, Jansson 2014, Martin 2012, Martin 2015, Mikkelsgard 2014, Ramon 2011, Reagon 2011, Secker 2012
**Finding 20:** Participants felt that being immersed in a social atmosphere offered them the opportunity to develop or practice social skills	
**Assessment for each CERQual component**	
Methodological limitations	Moderate concerns due to risk of recall bias in one study, poor reporting of data collection and sampling in one study and discussion of reflexivity
Coherence	Minor or no concerns regarding coherence
Relevance	Substantial concerns due to narrow range of settings (two studies in Norway and one study in UK)
Adequacy	Minor o no concerns regarding adequacy
**Overall CERQual assessment**	
Low confidence	Due to moderate concerns about methodological limitations and substantial concerns about relevance
**Contributing studies**	Boycott 2015, Haugli 2011, Kalef 2014
**EXPERIENCE OF PARTICIPATING IN A GROUP WITH OTHER PERSONS WITH CHRONIC HEALTH CONDITIONS**	
**Finding 21:** Several subjects attending an educational or support group found that meeting other persons who face similar problems made them feel normal and less isolated	
**Assessment for each CERQual component**	
Methodological limitations	Moderate concerns regarding poor reporting of representativeness of sample and poor discussion of reflexivity
Coherence	No or minor concerns regarding coherence
Relevance	Minor concerns regarding relevance (four studies in three Nordic countries, two studies in UK, one in France; different diseases; different strategies)
Adequacy	No or minor concerns regarding adequacy
**Overall CERQual assessment**	
Moderate confidence	Due to moderate concerns about methodological limitations and minor concerns about relevance

**Contributing studies**	Andersen 2014, Cameron 2012, De Blassi 2014, Glavare 2012, Haugli 2011, Jansson 2014, Secker 2012
**Finding 22:** Meeting persons with similar concerns helped participants to learn more about their situation and motivated them to try a new approach	
**Assessment for each CERQual component**	
Methodological limitations	Moderate concerns due to poor discussion of reflexivity, poor general reporting in one study and risk of recall bias in one study
Coherence	No or minor concerns regarding coherence
Relevance	Moderate concerns regarding relevance (two studies in Nordic countries, one study in UK, one study in France; different diseases, different strategies)
Adequacy	Minor concerns regarding adequacy due to thin data in two studies
**Overall CERQual assessment**	
Moderate confidence	Due to moderate concerns about methodological limitations and relevance, and minor concerns regarding adequacy
**Contributing studies**	De Blassi 2014, Haugli 2011, Jansson 2014, Secker 2012
**Finding 23:** Some persons experienced difficulties sharing personal experiences in a group	
**Assessment for each CERQual component**	
Methodological limitations	Serious concerns due to poor discussion of reflexivity, risk of recall bias in one study and poor general reporting in one study
Coherence	No or minor concerns regarding coherence
Relevance	Moderate concern about relevance due to limited settings (two studies in Nordic countries, one study in UK; most participants with MD; different strategies)
Adequacy	Minor concerns regarding adequacy
**Overall CERQual assessment**	
Low confidence	Due to serious concerns about methodological limitations, moderate concerns about relevance and minor concerns about adequacy
**Contributing studies**	Haugli 2011, Jansson 2014, Reagon 2011
**EXPERIENCE AT THE WORKPLACE**	

**Finding 24:** Support from employers and managers is perceived by employees as a key element to succeed in their professional integration	
**Assessment for each CERQual component**	
Methodological limitations	Moderate concerns regarding poor reporting of representativeness of sample and poor discussion of reflexivity
Coherence	No or minor concerns regarding coherence
Relevance	Moderate concerns (five studies in two Nordic countries, two studies in UK, one in Belgium; different diseases; different strategies)
Adequacy	No or minor concerns regarding adequacy
**Overall CERQual assessment**	
Moderate confidence	Due to moderate concerns about methodological limitations and relevance
**Contributing studies**	Areberg 2013, Cameron 2012, Glavare 2012, Haugli 2011, Jansson 2014, Mikkelsgard 2014, Lewis 2013, Tiedtke 2012
**Finding 25:** The amount of support provided by colleagues plays an important role in work integration	
**Assessment for each CERQual component**	
Methodological limitations	Moderate concerns due to poor reporting of representativeness of the sample in three studies, data collection in one study and data analysis in one study
Coherence	No or minor concerns regarding coherence
Relevance	Moderate concerns due to limited settings (two studies in UK, one study in Sweden; different diseases; different strategies)
Adequacy	No or minor concerns regarding adequacy
**Overall CERQual assessment**	
Moderate confidence	Due to moderate concerns about methodological limitations and relevance
**Contributing studies**	Cameron 2012, Glavare 2012, Lewis 2013
**Finding 26:** Some organizations have a working culture and physical environment that makes the provision of support easier	
**Assessment for each CERQual component**	

Methodological limitations	Moderate concerns due to poor reporting of representativeness of sample, data collection and poor discussion of reflexivity
Coherence	No or minor concerns about coherence
Relevance	Minor concerns regarding relevance (two studies in Nordic countries, two in IK, one in Belgium, one in different European countries; most participants with MD; different strategies)
Adequacy	No or minor concerns regarding adequacy
**Overall CERQual assessment**	
Moderate confidence	Due to moderate concerns about methodological limitations and relevance
**Contributing studies**	Cameron 2012 , Kalef 2014, Jansson 2014, Lewis 2013, Nieminen 2012, Vandekinderen 2012
**MOTIVATING ASPECTS OF WORK**	
**Finding 27:** The expectation to improve their financial situation motivated people with chronic health conditions to find a job	
**Assessment for each CERQual component**	
Methodological limitations	Moderate concerns regarding poor reporting of sample, data collection and poor discussion of reflexivity
Coherence	No or minor concerns regarding coherence
Relevance	Moderate concerns due to narrow range of settings (two studies in Nordic countries, one study in UK, one in Belgium; all participants unemployed; most of them with MD; strategy in three of four studies: supported employment)
Adequacy	No or minor concerns about adequacy
**Overall CERQual assessment**	
Moderate confidence	Due to moderate concerns about methodological limitations and relevance
**Contributing studies**	Hasson 2011, Lewis 2013, Mikkelsgard 2014, Vandekinderen 2012
**Finding 28:** Subjects found that having a job or doing an internship facilitates social inclusion and interaction	
**Assessment for each CERQual component**	

Methodological limitations	Moderate concerns due to poor reporting of sampling, sample characteristics and data analysis, and poor discussion of reflexivity
Coherence	No or minor concerns regarding coherence
Relevance	Moderate concerns due to narrow range of settings (two studies in Norway, one study in UK, one in different European countries; all participants unemployed; different diseases; strategy in two studies supported employment and in two studies job training)
Adequacy	No or minor concerns about adequacy
**Overall CERQual assessment**	
Moderate confidence	Due to moderate concerns about methodological limitations and relevance
**Contributing studies**	Kalef 2014, Lewis 2013, Mikkelsgard 2014, Nieminen 2012
**Finding 29:** Having an occupation allowed persons with chronic diseases to focus on something other than the disease	
**Assessment for each CERQual component**	
Methodological limitations	Moderate concerns due to poor reporting in general in one study and poor reporting of representativeness of sample in two studies
Coherence	No or minor concerns regarding coherence
Relevance	Moderate concerns about relevance due to limited variety of settings (two studies in Nordic countries, one study in different European countries; all participants unemployed; all with MD; the strategy in two studies was employment support and in one study job training)
Adequacy	Moderate concerns regarding adequacy due to one study with thin data
**Overall CERQual assessment**	
Low confidence	Due to moderate concerns about methodological limitations, relevance and adequacy
**Contributing studies**	Areberg 2013, Mikkelsgard 2014, Ramon 2011
**Finding 30:** Participants found it very important to do something that they could enjoy	
**Assessment for each CERQual component**	
Methodological limitations	Serious concerns due to poor general reporting in one of two studies

	and risk of recall bias in the second study
Coherence	Moderate concerns due poor presentation of results in one of two studies
Relevance	Serious concerns due to only two studies (one in UK, one in different European countries; all participants with MD; all unemployed when receiving the strategy)
Adequacy	Serious concerns due to thin data in one of two studies
**Overall CERQual assessment**	
Very low confidence	Due to serious concerns about methodological limitations, relevance and adequacy, and moderate concerns due to coherence
**Contributing studies**	Boycott 2015, Ramon 2011
**Finding 31:** Having a job was associated with an increase in self-esteem and self-confidence	
**Assessment for each CERQual component**	
Methodological limitations	Moderate concerns due to poor general reporting in one study and poor reporting regarding representativeness of sample in two studies
Coherence	No or minor concerns regarding coherence
Relevance	Moderate concerns due to limited settings (one study in Norway, one study in UK, one in different European countries; most participants with MD; all unemployed when receiving the strategy; two of three studies: supported employment)
Adequacy	Minor concerns due to thin data in one of three studies
**Overall CERQual assessment**	
Low confidence	Due to moderate concerns about methodological limitations and relevance, and minor concerns regarding adequacy
**Contributing studies**	Lewis 2013, Mikkelsgard 2014, Nieminen 2012

References of contributing studies

Andersen, M.F.; Nielsen, K.; Brinkmann, S. How do workers with common mental disorders experience a multidisciplinary return-to-work intervention? A qualitative study. Journal of Occupational Rehabilitation 2014, 24, 709-724.

Areberg, C.; Bjorkman, T.; Bejerholm, U. Experiences of the individual placement and support approach in persons with severe mental illness. Scandinavian Journal of Caring Sciences 2013, 27, 589-596.

Boycott, N.; Akhtar, A.; Schneider, J. "Work is good for me": Views of mental health service users seeking work during the uk recession, a qualitative analysis. Journal of Mental Health 2015, 24, 93-97.

Buus, N.; Jensen, L.D.; Maribo, T.; Gonge, B.K.; Angel, S. Low back pain patients' beliefs about effective/ineffective constituents of a counseling intervention: A follow-up interview study. Disability & Rehabilitation 2015, 37, 936-941.
Cameron, J.; Walker, C.; Hart, A.; Sadlo, G.; Haslam, I.; Retain Support, G. Supporting workers with mental health problems to retain employment: Users' experiences of a uk job retention project. Work 2012, 42, 461-471.
De Blasi, G.; Bouteyre, E.; Bretteville, J.; Boucher, L.; Rollin, L. Multidisciplinary department of "return to work after a cancer": A french experience of support groups for vocational rehabilitation. Journal of Psychosocial Oncology 2014, 32, 74-93.
Germundsson, P.; Hillborg, H.; Danermark, B. Interagency collaboration in vocational rehabilitation for persons with mental health problems: The perspective of the service users and the professionals. Disability & Society 2011, 26, 699-713.
Glavare, M.; Lofgren, M.; Schult, M.L. Between unemployment and employment: Experience of unemployed long-term pain sufferers. Work 2012, 43, 475-485.
Hasson, H.; Andersson, M.; Bejerholm, U. Barriers in implementation of evidence-based practice: Supported employment in swedish context. Journal of Health Organization & Management 2011, 25, 332-345.
Haugli, L.; Maeland, S.; Magnussen, L.H. What facilitates return to work? Patients' experiences 3 years after occupational rehabilitation. Journal of Occupational Rehabilitation 2011, 21, 573-581.
Higgins, A.; Porter, S.; O'Halloran, P. General practitioners' management of the long-term sick role. Social Science & Medicine 2014, 107, 52-60.
Hubertsson, J.; Petersson, I.F.; Arvidsson, B.; Thorstensson, C.A. Sickness absence in musculoskeletal disorders - patients' experiences of interactions with the social insurance agency and health care. A qualitative study. BMC Public Health 2011, 11, 107.
Jansson, I.; Perseius, K.I.; Gunnarsson, A.B.; Bjorklund, A. Work and everyday activities: Experiences from two interventions addressing people with common mental disorders. Scandinavian Journal of Occupational Therapy 2014, 21, 295-304.
Kalef, L.; Barrera, M.; Heymann, J. Developing inclusive employment: Lessons from telenor open mind. Work 2014, 48, 423-434.
Lewis, R.; Dobbs, L.; Biddle, P. 'If this wasn't here i probably wouldn't be': Disabled workers' views of employment support. Disability & Society 2013, 28, 1089-1103.
Martin, M.H.T.; Moefelt, L.; Nielsen, M.B.D.; Rugulies, R. Barriers and facilitators for implementation of a return-to-work intervention for sickness absence beneficiaries with mental health problems: Results from three danish municipalities. Scandinavian Journal of Public Health 2015, 43, 423-431.
Martin, M.H.T.; Nielsen, M.B.D.; Petersen, S.M.A.; Jakobsen, L.M.; Rugulies, R. Implementation of a coordinated and tailored return-to-work intervention for employees with mental health problems. Journal of Occupational Rehabilitation 2012, 22, 427-436.
Mikkelsgard, K.A.; Granerud, A.; Hoye, S. People with mental illness returning to work: A qualitative evaluation of a norwegian project. Scandinavian Journal of Occupational Therapy 2014, 21, 172-180.
Nieminen, I.; Ramon, S.; Dawson, I.; Flores, P.; Leahy, E.; Pedersen, M.L.; Kaunonen, M. Experiences of social inclusion and employment of mental health service users in a european union project. International Journal of Mental Health 2012, 41, 3-23.
Ramon, S.; Griffiths, C.A.; Nieminen, I.; Pedersen, M.; Dawson, I. Towards social inclusion through lifelong learning in mental health: Analysis of change in the lives of the emilia project service users. International Journal of Social Psychiatry 2011, 57, 211-223.
Reagon, C. Vocational rehabilitation in wales: A mixed method evaluation of condition management programmes. International Journal of Rehabilitation Research 2011, 34, 22-28.
Secker, J.; Pittam, G.; Ford, F. Customers' perspectives on the impact of the pathways to work condition management programme on their health, well-being and vocational activity. Perspectives in public health 2012, 132, 277-281.

Tiedtke, C.; Donceel, P.; Knops, L.; Désiron, H.; de Casterlé, B.D.; de Rijk, A. Supporting return-to-work in the face of legislation: Stakeholders' experiences with return-to-work after breast cancer in belgium. Journal of Occupational Rehabilitation 2012, 22, 241-251.
Vandekinderen, C.; Roets, G.; Vandenbroeck, M.; Vanderplasschen, W.; Van Hove, G. One size fits all? The social construction of dis-employ-abled wo
